# Transcriptome Analysis of Epigenetically Modulated Genome Indicates Signature Genes in Manifestation of Type 1 Diabetes and Its Prevention in NOD Mice

**DOI:** 10.1371/journal.pone.0055074

**Published:** 2013-01-30

**Authors:** Sundararajan Jayaraman, Akshay Patel, Arathi Jayaraman, Vasu Patel, Mark Holterman, Bellur Prabhakar

**Affiliations:** 1 Department of Surgery, University of Illinois at Chicago, Chicago, Illinois, United States of America; 2 Department of Microbiology & Immunology, University of Illinois at Chicago, Chicago, Illinois, United States of America; Hemocentro de Ribeirão Preto, HC-FMRP-USP., Brazil

## Abstract

Classic genetic studies implicated several genes including immune response genes in the risk of developing type 1 diabetes in humans. However, recent evidence including discordant diabetes incidence among monozygotic twins suggested a role for epigenetics in disease manifestation. NOD mice spontaneously develop type 1 diabetes like humans and serve as an excellent model system to study the mechanisms of type 1 diabetes as well as the efficacy of maneuvers to manipulate the disease. Using this preclinical model, we have recently demonstrated that pharmacological inhibition of histone deacetylases can lead to histone hyperacetylation, selective up-regulation of interferon-γ and its transactivator *Tbx21/Tbet*, and amelioration of autoimmune diabetes. In the current study, we show that chromatin remodeling can render splenocytes incapable of transferring diabetes into immunodeficient NOD.*scid* mice. To elucidate the underlying mechanisms of drug-mediated protection against type 1 diabetes, we performed global gene expression profiling of splenocytes using high throughput microarray technology. This unbiased transcriptome analysis unraveled the exaggerated expression of a novel set of closely related inflammatory genes in splenocytes of acutely diabetic mice and their repression in mice cured of diabetes by chromatin remodeling. Analysis of gene expression by qRT-PCR using RNA derived from spleens and pancreata of cured mice validated the suppression of most of these genes, indicating an inverse correlation between the high levels of these inflammatory genes and protection against diabetes in NOD mice. In addition, higher-level expression of genes involved in insulin sensitivity, erythropoiesis, hemangioblast generation, and cellular redox control was evident in spleens of cured mice, indicating their possible contribution to protection against type 1 diabetes. Taken together, these results are consistent with the involvement of epistatic mechanisms in the manifestation of autoimmune diabetes and further indicate the utility of chromatin remodeling in curing this complex autoimmune disorder.

## Introduction

Type 1 diabetes (T1D) is the most common childhood autoimmune disease studied for decades but the etiology of this complex disorder remains obscure. Classic genetic approaches including genome wide association studies and single nucleotide polymorphism analysis indicated a strong association between T1D in man and the major histocompatibility complex (MHC) encoded class I and II alleles [1]. In addition, several non-MHC genes including *INS* (insulin), *PTPN22* (lymphoid tyrosine phosphatase protein), *CTLA4* (cytotoxic T-lymphocyte-associated antigen 4), *IL2RA* (interleukin 2 receptor α), *STAT3*, *STAT4*, *IL10*, *IL19*, *IL20*, *IL27*, and *CD69* have been implicated in the development of T1D [2]. However, the mechanisms by which these genes contribute to diabetes susceptibility remain unknown. Availability of non-obsese diabetic (NOD) mice and their congenic variants has advanced our understanding of the genes involved in T1D. The major contributor of diabetes susceptibility in NOD mice is the MHC (H-2^g7^), designated as *Idd1* locus on chromosome 17 [3]. Although homozygous expression of the susceptible MHC haplotype (K^d^, A^g7^, E^null^, and D^b^) is required for high penetrance, this alone is not sufficient to cause T1D [4]. Interestingly, the expression of H2^nb1^ (K^d^, A^nb1^, E^k^, and D^b^) on dendritic cells and macrophages but not on B cells afforded protection against T1D, indicating a role for tissue-specific non-MHC genes in protection against T1D [4]. However, the nature of the non-MHC genes expressed in accessory cells that contribute to protection against T1D remains unknown.

Several lines of evidence including enhanced T1D incidence among Caucasians living in Europe, discordant rate of T1D among monozygotic twin pairs, and lower incidence of T1D in some individuals harboring the ‘risk genes’ indicate that although the MHC genes may impart T1D susceptibility, they are not sufficient to cause the disease [5]–[6]. Epigenetics, heritable changed gene expression patterns that cannot be attributed to alteration in the DNA sequence, has been implicated in many diseases including cancer and diabetes [7]–[10]. One prominent epigenetic mechanism involves repression of gene transcription as a consequence of histone modification mediated by histone deacetylases (HDAC) [11]. It is well established that small molecule HDAC inhibitors including Trichostatin A (TSA) can alter gene transcription and ameliorate a number of diseases including cancers and other diseases in experimental models [12]–[13]. Consistently, we have demonstrated that TSA treatment can prevent the manifestation of T1D in NOD mice, associated with histone H3 hyperacetylation, and selective up-regulation of genes encoding CD4^+^ T-cell-derived lymphokine, *Ifng* and its transcription factor, *Tbet/Tbx21* [8]. Inasmuch as complex disorders like T1D are likely to be regulated by epistatic mechanisms [9], it is important to understand the nature of genes regulated by chromatin remodeling that can potentially contribute to protection against T1D.

Transcriptome (gene expression profiling) analyses have unraveled new transcriptional alterations in several diseases. High throughput microarray technology has been used to understand the changes in gene expression in the pancreas during the natural course of T1D in NOD mice [14]. Global gene expression profiling was also performed on un-activated peripheral lymphoid tissues as well as activated CD4^+^ T-cells derived from NOD mice and diabetes resistant NOD congenic strains, as well as prediabetic NOD mice immunized with glutamic acid decarboxylase peptide to protect against diabetes [15]–[20]. Although these studies unraveled the gene expression profiles of lymphoid tissues during the prediabetic stage, little is known about the gene expression profiles during full-blown diabetes and importantly, how they can be influenced by epigenetic regulation. The results presented herein indicate that epigenetic modulation of the genome can result in the ablation of diabetogenic potential of splenocytes in diabetes-prone NOD mice. To elucidate the genes possibly involved in diabetes pathogenesis and protection against it, we analyzed the global gene expression profiles of spleens as in previous studies [15]–[20]. Since spleen is the major peripheral lymphoid organ and contains T-cells capable of transferring T1D into immuodeficient NOD.*scid* mice [21], it is the logical choice of investigational material for the understanding of the epigenomics of T1D. Our data indicate that chromatin remodeling resulted in simultaneous down-regulation of a set of inflammatory genes and up-regulation of a number genes involved in a variety of key cellular functions, including glucose homeostasis. These data are consistent with the contention that complex disorder like T1D involves differential contribution of a variety of genes that participate in multiple signaling and metabolic pathways.

## Materials and Methods

### Mice and Diabetes Assessment

This study was carried out in strict accordance with the recommendations in the Guide for the Care and Use of Laboratory Animals of the National Institutes of Health. The protocol was approved by the Committee on the Ethics of Animal Experiments of the University of Illinois at Chicago (Animal Welfare Assurance Number: A3460-01).

Female NOD/Ltj (H-2^g7^) mice (Jackson Laboratories, Bar Harbor, ME) that were diabetes free by 18 wk of age were injected s.c with TSA (500 µg/Kg body weight) at weekly intervals between 18 and 24 wk of age [8]. Non-fasting blood glucose levels were monitored weekly and >250 mg/dL for two consecutive weeks were considered diabetic [8], [22]. Whereas overtly diabetic mice were killed between 24 and 28 wk of age, TSA-treated and cured mice were killed between 28 and 34 wk of age and spleens and pancreata harvested. Histological analysis of paraffin embedded pancreata was performed as described earlier [22]. Each NOD.*scid* mouse was injected i.v with 2×10^7^ splenocytes obtained from individual diabetic or cured mice. Peripheral blood glucose levels were monitored at weekly intervals to determine diabetes induction.

### Global Gene Expression Profiling

Overtly diabetic mice were killed between 24 and 28 wk of age. Only mice that were cured of T1D by TSA treatment were killed between 28 and 34 wk of age. TSA-treated mice that remained diabetic were not included in the analysis. Total RNA was extracted from individual spleens after lysing in TRIzol, as described [8], [22]. RNA from 4 to 6 mice per group was pooled to minimize the expression bias. RNA was further purified on RNeasy columns (Qiagen, Valencia, CA) and the integrity of RNA was assessed by formaldehyde agarose gel electrophoresis and BioRad Experion Bioanalyzer. Samples were analyzed in duplicate using the Affymetrix GeneChip Mouse Genome 430 2.0 microarray (Santa Clara, CA) that contained 45,000 probe sets, representing 34,000 mouse genes. Labeling and hybridizations were performed at the Genomics Core Facility of the University of Illinois at Chicago, according to the recommended protocols by Affymetrix. In brief, double-stranded cDNA was made from 1–5 µg of total cellular RNA. Each sample was *in vitro* transcribed in the presence of biotinylated dNTPs (Enzo Diagnostics, Farmingdale, NY) in duplicate. Biotinylated cRNA was hybridized to microarrays and scanned. Each array was analyzed for total background, raw noise, average signal present, signal intensity of species-specific house-keeping genes, relative signal intensities of labeling controls, absolute signal intensities of hybridization controls, and GCOS scale factors. All 6 Affymetrix labeling reactions and 6 Affymetrix GeneChip Mouse Genome 430 2.0 hybridizations passed quality criteria. Data were analyzed using the ‘S-Plus’ 6.2 statistical package and ‘S+Array Analyzer’ v2.0.1 from Insightful, normalized by quantiles and summarized using the Robust Multi-array Average method. T-test was used to identify significant, differentially expressed transcripts. Raw LPE test p-values were corrected for False Discovery Rate by Benjamini-Hochberg procedure with a statistical threshold of p-value <0.001. ANOVA was used to identify statistically significant, differentially expressed transcripts. Microarray datasets were deposited at Gene Expression Omnibus (accession no. GSE 26461). A total of 3,233 statistically significant, differentially expressed probe sets were identified in any comparison by ANOVA. Differentially expressed probe sets between untreated-diabetic and untreated-non-diabetic were 1,307; between cured and untreated-non-diabetic mice were 2,991; and 164 between untreated-diabetic and cured mice. Differentially expressed transcripts were annotated using the NetAffx Analysis Center (http://www.affymetrix.com) according to the Gene Ontology Database (http://www.geneontology.org/). Data clustered were filtered by an ANOVA p-value <0.05, and having a gene expression level of more than 10 in at least two hybridizations. A total of 164 probe sets met these criteria and were annotated according to Affymetrix’s “NetAffx Analysis Center.” Hierarchical clustering was performed on these 164 genes using ‘Average’ weighting method and Euclideal distance metric. The heat map is available as Supporting Information (Figure S1). Further biological annotation was performed using the DAVID web-based functional annotation tool (http://david.abcc.ncifcrf.gov) [23]. The resulting filtered 134 differentially expressed genes were subjected to Gene Ontology analysis.

### Validation of Selected Microarray Data

Quantitative reverse transcriptase polymerase chain reaction (qRT-PCR) was performed according to MIQE guidelines. Total RNA was extracted from un-induced splenocytes and those stimulated with immobilized anti-CD3 antibody for 18 h as described earlier [8], [22]. Pancreata were stored in RNALater (Life Technologies, Grand Island, NY) at −80°C and RNA was extracted from pancreata dissociated in TRIzol. Total RNA was treated with DNase using TURBO DNA-free kit (Life Technologies) and converted to cDNA using High-Capacity cDNA Reverse Transcription kit (Life Technologies). Genes of interest were amplified on an Applied Biosystems ViiA7 Real-Time PCR system (Life Technologies) using 1 µl of cDNA equivalent to 100 ng of RNA and 2X SYBR Premix Ex Taq (Perfect Real Time) reagent (Takara-Clontech, Mountain View, CA). No template control was included in qRT-PCR for each gene analyzed to monitor primer-dimer formation and omission of reverse transcriptase during cDNA synthesis was used to determine genomic DNA contamination in RNA preparations. In addition to melting curve analysis at the end of PCR amplification, amplicons were also analyzed on a 4% low melt agarose gel to verify the expected sizes of amplicons and the lack of primer-dimer. The primer sets for mouse *Gapdh* were described earlier [8], [22] and additional primer sets that were validated and used in this study are listed in Table S1. The Taqman probe for mouse *Mif* and all other primer sets were purchased from IDT (Coralville, IA). Each sample was analyzed in triplicate and the level of expression of genes of interest was determined using *Gapdh* as the normalizer and the 2^−ΔΔ*CT*^ method, as described [8], [22].

### Statistical Analyses

Data were analyzed for statistical significance using an unpaired two-tailed Student’s *t* test (GraphPad Prism 4.0c, San Diego, CA). Venn diagram was drawn using 3Venn aplet accessed at: http://theory.cs.uvic.ca/venn/EulerianCircles. GeneMania fast gene function predictions (version 2.7.12) were performed by accessing at www.genemania.org. Principal component analysis was performed using XLSTAT Version 2011.4.02 (www.xlstat.com).

## Results

### Epigenetic Modulation Ablates the Diabetogenic Potential of T-cells

**Figure 1:**
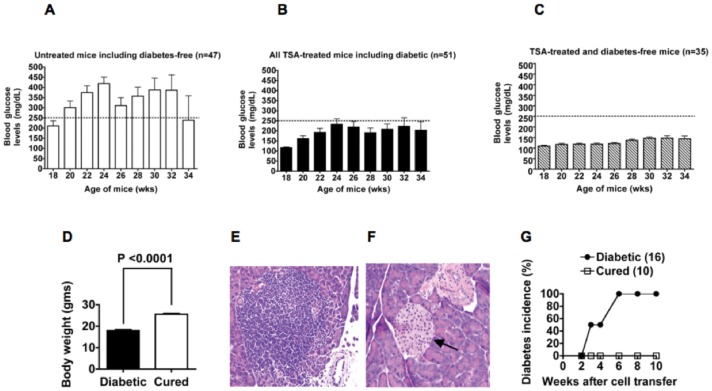
Epigenetic regulation of T1D. (**A**) The average blood glucose levels of 47 untreated female NOD mice are shown. Whereas a majority (80%) of untreated mice became diabetic (>250 mg/dL of blood glucose as indicated by the dotted line) when they reached >18 wk of age, only a minority (20%) of untreated mice remained non-diabetic till the end of the observation period, 34 wk. This is reflected by the lower non-fasting blood glucose levels at 34 wk of age in the small number of untreated mice. (**B**) Glycemic profiles of female mice treated with TSA during 18–24 wk of age indicate that a vast majority (71%) of these mice was protected from diabetes. However, the overall average blood glucose levels of these mice appeared to be higher than those found in younger (18–20 wk old) mice. This was attributed to the inclusion of higher levels of glucose in 29% of diabetic mice also in the analysis. (**C**) Depiction of glucose levels in TSA-treated mice that remained diabetes-free. Note that the levels of non-fasting glucose levels of these mice were similar to those of younger (18–20 wk old), non-diabetic mice. (**D**) Shown are changes in body weight of acutely diabetic mice and those treated with TSA (*n* = 35) at the time of sacrifice (32–34 wk of age). (**E**) Pancreatic section from overtly diabetic mice shows heavy infiltration of islets (x 40 magnification, Hematoxylin & Eosin staining). (**F**) Pancreata of cured mice typically contain smaller islets (indicated by an arrow) adjacent to blood vessels without accompanying inflammation (x 40 magnification). (**G**) Splenocytes were harvested individually from acutely diabetic mice and those cured of diabetes and transferred into individual NOD.*scid* mice. Diabetes was monitored weekly and the numbers of mice investigated are shown in parentheses.

A majority of adult female NOD mice develop T1D spontaneously, which serve as an excellent model for studying intervention strategies for this complex autoimmune disorder [8], [22], [24]. Consistently, data shown in Figure 1A, indicate that most (80%) of the untreated female NOD mice developed diabetes when they reached >18 wk of age, as indicated by the levels of non-fasting blood glucose higher than 250 mg/dL. Overtly diabetic mice had to be humanely sacrificed and the remaining 20% of mice were diabetes free at the end of the observation period, 34 wk, which is indicated by the lower levels of blood glucose (<250 mg/dL) in surviving mice (Figure 1A). Mice that did not develop diabetes by 18 wk of age were given weekly injections of TSA until the age of 24 wk. Treatment with TSA prevented the development of overt diabetes in a majority (69%) of mice [8] (Figure 1B). However, when the levels of blood glucose from TSA-treated mice including 29% of mice that failed to respond to treatment were averaged, the non-fasting blood glucose levels appeared to be higher than those of younger (18–20 wk old) non-diabetic mice, albeit below the 250 mg/dL cut-off level (Figure 1B). The glucose profiles of individual mice that were treated with TSA and remained free of diabetes demonstrated the maintenance of normoglycemia in all of them [8]. Consistently, when only the blood glucose levels of drug treated and diabetes-free mice were averaged, they were comparable to those of younger, non-diabetic mice (Figure 1C). These results indicate that TSA treatment restored normoglycemia robustly in a vast majority of mice and yet a small fraction of mice failed to respond to drug treatment and developed overt diabetes. We showed previously that instead of TSA, injection of mice with the vehicle, DMSO during 18 and 24 wk of age did not influence diabetes incidence [8]. Mice protected from T1D also gained body weight (Figure 1D) and lived longer, indicating one of the beneficial effects of restoring normoglycemia. Whereas the islets of Langerhans in the pancreas of overtly diabetic mice displayed invasive and heavy cellular infiltration (Figure 1 E), those of TSA treated and cured mice had minimal or no cellular infiltration (Figure 1F) [8]. Pancreata of cured mice contained distinctly smaller and well-defined islets adjacent to blood vessels, consistent with possible neogeneration of islets in drug treated mice.

To determine whether TSA treatment could diminish or abolish the ability of T-cells to mediate diabetes, splenocytes from cured mice were transferred into histocompatible but immunodeficient NOD.*scid* mice, which do not develop T1D due to the lack of T-cells and functional macrophages [21]. Whereas adoptive transfer of splenocytes from overtly diabetic mice induced diabetes, splenocytes derived from TSA-treated mice failed to transfer the disease into NOD.*scid* mice (Figure 1G), indicating the attenuation of the diabetogenic potential of T lymphocytes by epigenetic modulation of the genome.

### Chromatin Remodeling Altered Global Gene Expression

Previously, we have shown that TSA treatment alleviated T1D in NOD mice and also up-regulated the expression of the transcription factor *Tbx21/Tbet* and *Ifng* genes without altering the levels of some of the genes implicated in diabetes such as *Il4*, *Il17*, *Il18*, and *Tnfa* in activated T lymphocytes [8]. These data are consistent with discrete influence of chromatin remodeling on the transcriptional program of T-cells. Genome wide expression analysis, a non-hypothesis driven approach is ideal for analyzing the expression levels of thousands of genes simultaneously without bias. As in previous studies [15]–[20], we interrogated the transcriptome analysis of spleen since it is the major site of immune responses and contains both T-cells capable of transferring diabetes into immunodeficient NOD.*scid* mice [21] and macrophages that act as effectors of CD4^+^ T-cell mediated T1D [25]. Therefore, using high-throughput microarray technology, we determined the changes in global gene expression in un-induced splenocytes of 24–28 wk old, overtly diabetic mice and compared them with that of 28–34 wk old, TSA-treated and cured mice, as well as age-matched untreated-non-diabetic mice. Total RNA was extracted from individual spleens of untreated-non-diabetic mice, untreated overtly diabetic mice, and TSA-treated and cured mice. RNA was converted into cRNA, and used for hybridization with Affymetrix GeneChip Mouse Genome 430 2.0 microarrays that contain 45,000 probe sets representing 34,000 mouse genes. Three way analysis of gene expression in untreated-non-diabetic mice, overtly diabetic mice, and those cured by TSA treatment yielded 164 differentially regulated genes, filtered by an ANOVA p-value <0.05 (Table S2). These highly regulated genes were used for hierarchical clustering, which is available as supporting material (Figure S1).

**Figure 2:**
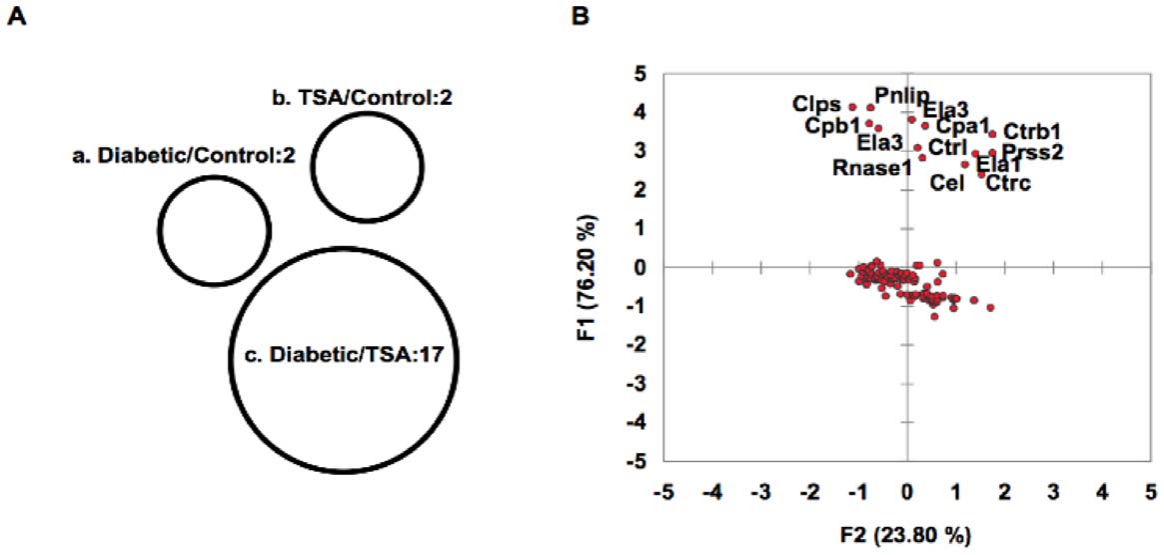
Genome wide expression profiling of splenocytes. (**A**) Differentially expressed 164 genes obtained from the microarray analysis were further analyzed. The area-proportional Venn diagram depicts 17 genes that were over-expressed in diabetic mice in comparison to TSA-treated mice. (**B**) Principal component analysis of diabetic/TSA-treated (F1) group indicates significant up-regulation of the same set of genes (*Clps, Pnlip, Cpb1, Ela3, Cpa1, Ctrb1, Ctrl, Prrs2, Rnase1, Ela1, Cel,* and *Ctrc*) depicted in Venn diagram (group c in **A**).

**Figure 3:**
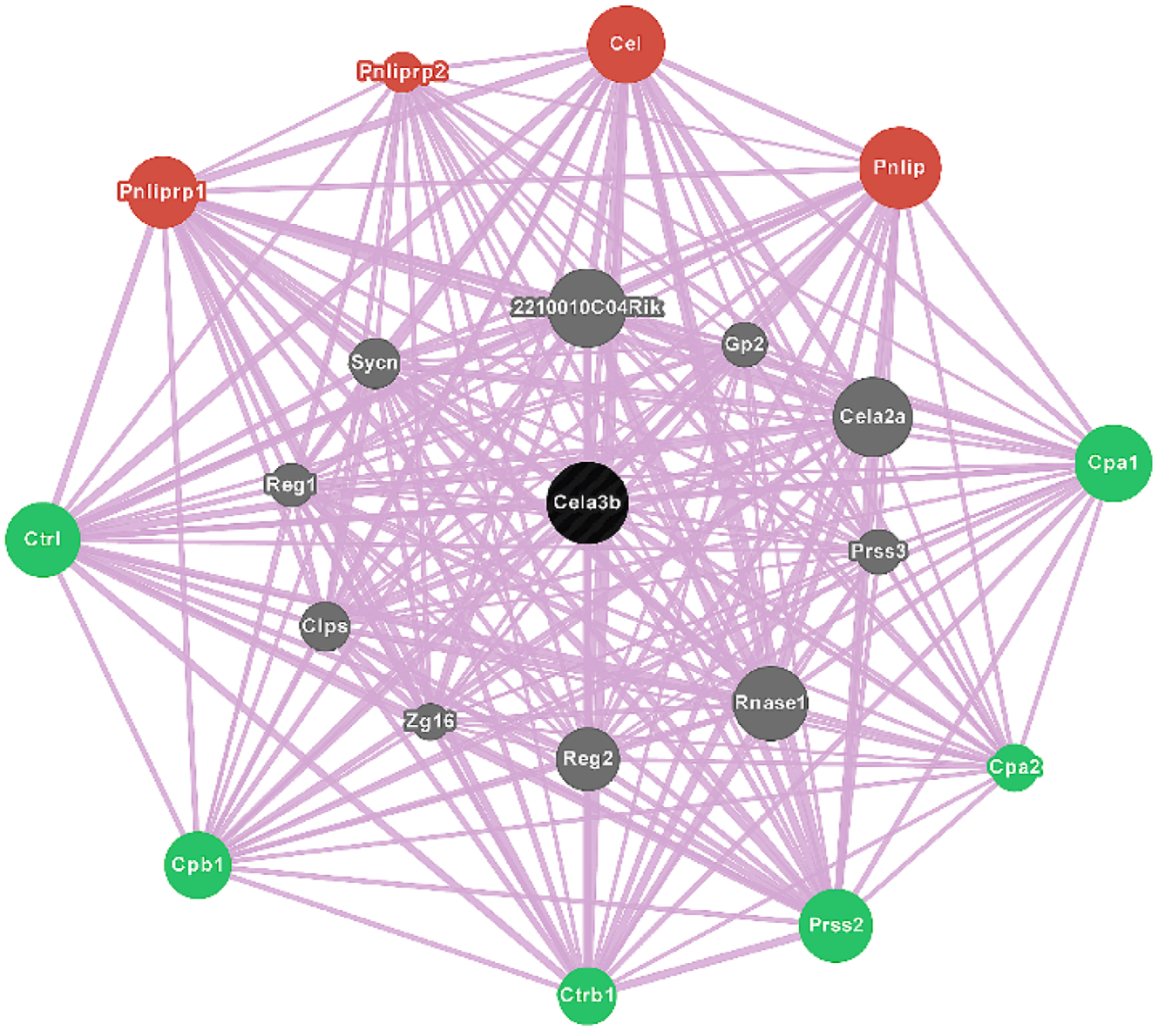
Epigenetic regulation of genes. GeneMANIA fast gene function predictions indicate the interrelationships between highly expressed genes in diabetic mice. The queried gene (black node), *Cela3b* is co-localized with numerous genes that share similar characteristics, including *Cela2a*, *Prss3*, *Rnase1,* and *Sync* (grey nodes). Closely related peptidases, *Ctrl, Cpb1*, *Ctrb1*, *Prss2, Cpa2*, and *Cpa1* (green nodes) and lipases, *Pnliprp1, Pnliprp2, Cel*, and *Pnlip* (red nodes) are also depicted.

Gene ontology was analyzed on these 164 genes using the DAVID bioinformatics resources tool [23]. Among the filtered 134 genes (Table S2), 60 (44.7%) and 50 (37.3%) genes, respectively code for phosphoproteins and acetylases, whereas 23 (17.1%) encode hydrolases, and 20 (14.9%) code for proteolytic enzymes (Table S3). A sizable fraction of these genes code for cytoplasmic proteins (n = 32, 23.8%) and non-membrane bounded organelles (n = 24, 17.9%). Area-proportional Venn diagram indicated that a distinct, non-overlapping set of genes (n = 17) was over-expressed in spleens of acutely diabetic mice (Table S4), which were repressed by TSA treatment (Figure 2A). Principal component analysis validated the up-regulation of a set of pro-inflammatory genes in un-induced spleens of overtly diabetic mice, and their repression by chromatin remodeling (Figure 2B). Further analysis revealed that these pro-inflammatory genes are co-localized and interact with each other, indicating their close physical proximity and functional relationships (Figure 3). In addition to genes coding for lipases, *Pnliprp1, Pnliprp2, Cel*, and *Pnlip*, those encode peptidases such as *Ctrl*, *Cpb1*, *Ctrb1*, *Prss2, Cpa2, and Cpa1* constitute the constellation of genes that may impact T1D pathogenesis.

### Validation of Repressed Inflammatory Genes in Drug Treated Mice by qRT-PCR

**Figure 4:**
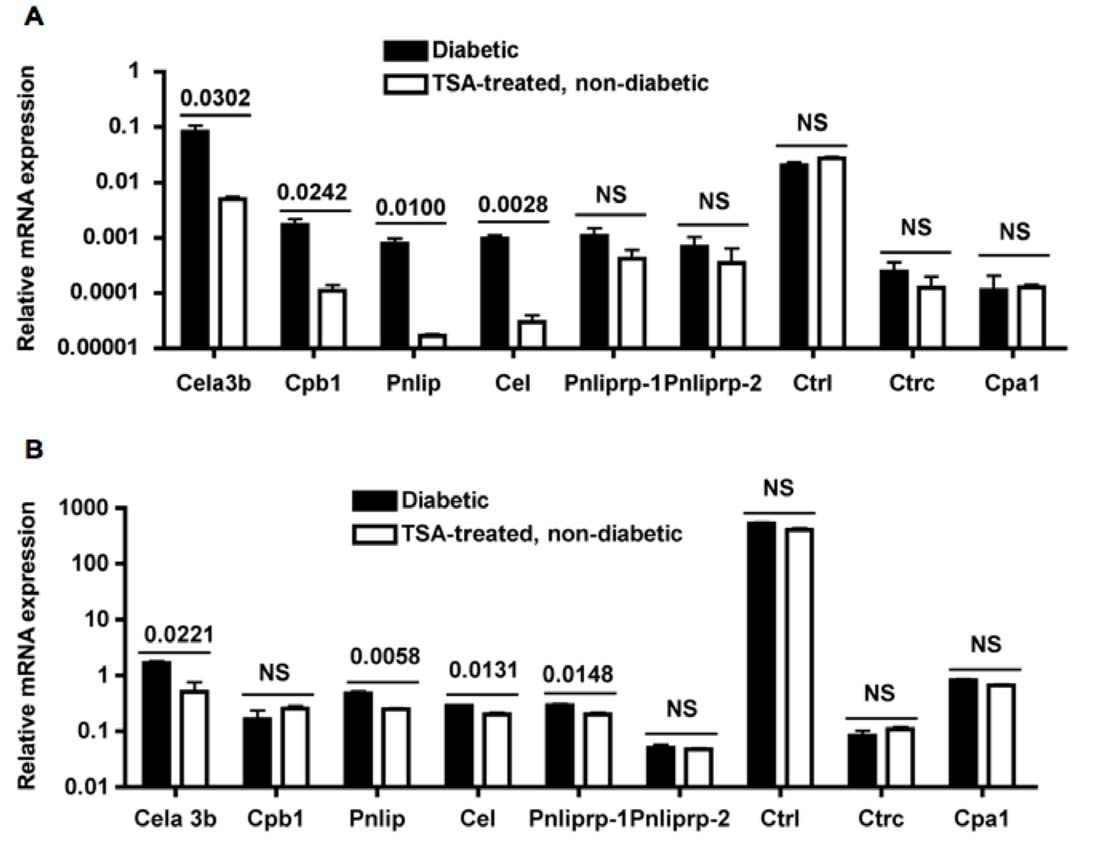
Repression of gene expression in splenocytes and pancreata by TSA treatment. (**A**) Total RNA was extracted from splenocytes of 24–28 wk old overtly diabetic mice and 28–34 wk old TSA-treated and cured mice that were different from those used for microarray analysis. RNA was converted into cDNA and used for validation of microarray data by qRT-PCR. (**B**) Expression of the same set of genes was determined by using RNA isolated from pancreata of diabetic and cured mice. Mean +/− SD of triplicate determinations from a representative of 2–3 experiments are shown. In each experiment, RNA was pooled from 3–5 mice. P values between groups are indicated. NS, not significant.

Expression levels of selected top hit genes were verified by qRT-PCR using primer sets that we validated following MIQE guidelines and RNA derived from spleens and pancreata of mice that were different from those used for microarray analysis. Treatment with TSA significantly suppressed the expression of 4 out of 9 genes tested, *Cela3b* (elastase 3), *Cpb1* (carboxyepetidase B1), *Pnlip* (pancreatic lipase), and *Cel* (carboxyl ester lipase) in spleens (Figure 4A). In addition to *Cela3b*, *Pnilp*, and *Cel, Pnliprp-1* (pancreatic lipase-related protein 1) was also repressed in the target organ, pancreas of cured mice (Figure 4B). However, *Ctrl* (chymotrypsin-like) gene substantially over-expressed in the exocrine pancreas [26] was not repressed by TSA treatment, indicating the selectivity of gene regulation by chromatin remodeling in the pancreata.

### Concurrent Exaggerated Expression of Genes Involved in Critical Cellular Functions by Chromatin Remodeling

**Figure 5:**
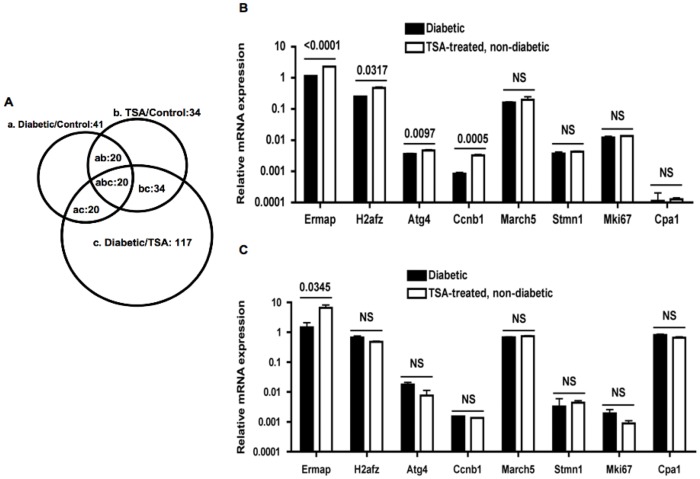
Exaggerated expression of genes in spleens and pancreata of TSA treated mice. (**A**) Area-proportional Venn diagram depicts the distribution of 117 over-expressed genes in TSA treated group (c). Small numbers of genes were also differentially expressed in other combinations (a and b). (**B**) A selected set of genes up-regulated following TSA treatment based on microarray hybridization signals was validated by qRT-PCR using RNA derived from splenocytes of diabetic and cured mice, as described in Figure 4. Total RNA was converted into cDNA and analyzed. (**C**) Gene expression was analyzed using RNA derived from pancreata of overtly diabetic mice and TSA-treated and cured mice. Each data point represents the mean +/− SD of triplicate determinations from a representative of 2–3 experiments. In each experiment, RNA was pooled from 3–5 mice. P values between groups are indicated. NS, not significant.

In tissue culture cell lines, treatment with HDAC inhibitors unleashed the transcription of ∼20% of genes [12]. Although an estimate of the proportion of genes that are up regulated *in vivo* by HDAC inhibitors is unknown, our microarray analysis indicated the up-regulation of a large number of genes (*n* = 117) in spleens by chromatin remodeling (Table S5). Venn diagram indicated that some of these genes were commonly up-regulated in untreated-non-diabetic mice as well (Figure 5A). We validated several of these highly up-regulated genes by qRT-PCR. Of note is the substantial increase in the expression of erythrocyte-specific gene, *Ermap* (erythroblast membrane-associated protein) [27] in both spleens (Figure 5B) and pancreata of cured mice (Figure 5C). However, other genes such as *H2afz* (histone H2A family member Z), important for proper gene expression and genome stability [28], *Ccnb1* (cyclin B1) involved in cell cycling [29], and *Atg4* (autophagy related 4A) gene, crucial for clearance of damaged cells [30], were up-regulated by TSA treatment only in spleens (Figure 5B) and not in the pancreas (Figure 5C).

**Figure 6:**
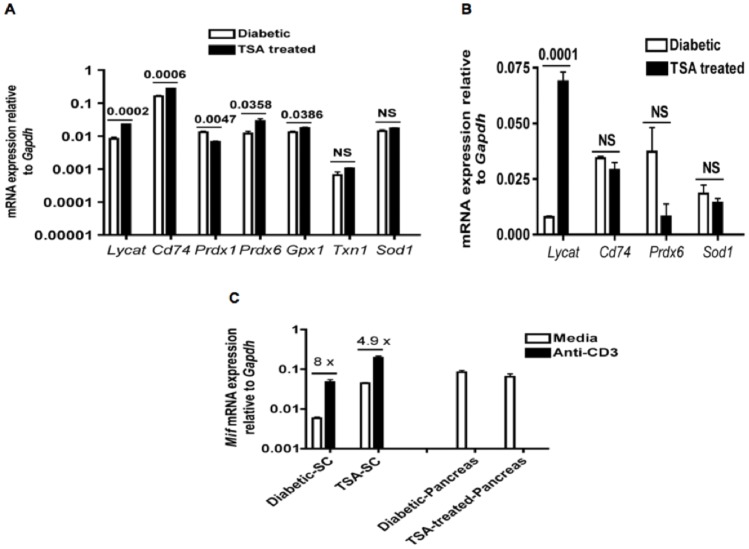
Modulation of genes implicated in various cellular functions by TSA treatment. (**A**) Total RNA was derived from splenocytes of diabetic and cured mice as indicated in Figure 4 & 5, and used for analyzing the expression of anti-oxidant genes and additional genes by qRT-PCR. (**B**) Total RNA derived from pancreata was probed similarly for the expression of indicated genes. (**C**) Splenic T lymphocytes from diabetic and cured mice were stimulated with immobilized anti-CD3, RNA extracted and analyzed for *Mif* expression. Steady state level expression of *Mif* was also analyzed in pancreata by qRT-PCR. Fold-increase in *Mif* gene expression is indicated. Each data point represents mean +/− SD of triplicate determinations. P values are given. NS indicates not significant. SC, splenocytes.

Interestingly, lysocardiolipin acyltransferase (*Lycat*), involved in the development of hematopoietic and endothelial lineages during embryogenesis [31], was enhanced in both spleens (Figure 6A) and pancreata of drug-treated mice (Figure 6B). The expression of the gene encoding the receptor of migration inhibition factor (MIF), CD74 was also enhanced in spleens (Figure 6A) but not in the pancreas of cured mice (Figure 6B). Insulin-producing β-cells are exquisitely sensitive to oxidant-induced injury and up-regulation of anti-oxidants can potentially alleviate diabetic complications [32]. Consistently, TSA treatment increased the expression of anti-oxidant genes, *Prdx6* (peroredoxin 6) and *Gpx1* (glutathione peroxidase 1), whereas *Prdx1* (peroredoxin 1) was decreased, and the levels of *Txn1* (thioredoxin 1) and *Sod1* (superoxide dismutase 1) remained unaltered in spleens (Figure 6A). In contrast, TSA treatment did not influence the expression of the anti-oxidant gene *Prdx6* or *Sod1* in pancreata (Figure 6B), suggesting the tissue-specific nature of the epigenetic regulation of anti-oxidant genes.

Treatment of NOD mice with TSA increased the steady state level expression of the gene encoding MIF, (*Mif*) in un-induced splenocytes in comparison to that of diabetic mice (Figure 6C). In order to determine whether epigenetic modulation can increase the inducible expression of *Mif* in T-cells, splenocytes were stimulated with immobilized anti-CD3 antibody and the transcript level was determined by qRT-PCR. Even though activation increased the expression of *Mif* in T-cells derived from diabetic spleens, higher level of *Mif* transcript was evident in activated T-cells from TSA-treated and cured mice (Figure 6C). In contrast, TSA treatment failed to alter the constitutive expression of *Mif* in pancreata. Thus, chromatin remodeling enhanced the expression of genes encoding MIF and its canonical receptor CD74 [33] in spleens but not in the target organ, pancreas (Figure 6A, B & C). Taken together, these data indicate that chromatin remodeling abrogated the diabetogenicity of T lymphocytes, accompanied by significant changes in gene expression in spleens and only with modest effects on the transcription of genes in pancreata.

## Discussion

Here we report for the first time, the results of the transcriptome analysis of overtly diabetic female NOD mice and age- and sex-matched mice that were rendered diabetes free by epigenetic modulation of the genome using a small molecule HDAC inhibitor, TSA. This is in contrast to previous transcriptome analyses of peripheral lymphoid tissues obtained from prediabetic, typically 4–12 wk old NOD mice, diabetes-resistant NOD congenic strains harboring the entire MHC class II or *Idd* loci from diabetes resistant B6 and B10 mice [15]–[19] as well as NOD mice immunized to prevent diabetes [20]. Since pancreata of prediabetic NOD mice are characterized by benign insulitis without aberrant glucose control [24], changes in gene expression documented in previous studies are relevant to the induction phase of the disease whereas those unraveled in our study pertain to the manifestation of full blown diabetes. Surprisingly, chromatin remodeling did not alter the expression of any of the “disease-associated” genes implicated in T1D, such as MHC class I and II loci, as well as non-MHC linked genes such as *Ins, Ctla4, Il2, Il2ra, Il21, and Ptpn22* [3]. These data suggest that although ‘susceptibility genes’ may predispose mice for diabetes development in an unknown manner, quantitative modification of these genes does not seem mandatory for the manifestation of full-blown diabetes later in life.

It is remarkable that the combination of microarray analysis and epigenetic modulation of the genome unraveled the exaggerated expression of a novel set of 17 closely related inflammatory genes in spleens of overtly diabetic NOD mice. We validated the higher expression of *Cel* (carboxyl ester lipase), *Cela3b* (chymotrypsin-like elastase family, member 3B), and *Pnlip* (pancreatic lipase) in both spleens and pancreata of overtly diabetic mice by qRT-PCR, gold standard for the quantification of gene expression. Similarly, previous microarray studies noted higher expression of carboxyl ester lipase (*Cel*) in spleens of prediabetic NOD mice when compared to diabetes-resistant strains of mice [15], [19]. Although these genes are highly expressed in the exocrine pancreas, they are also expressed in inflammatory cells such as macrophages and neutrophils under pathological conditions, including infections [34]–[36]. Since monocytes but not neutrophils are predominantly found in the inflammatory infiltrate of the islets [25], the pro-inflammatory genes identified in our study are likely to be expressed by monocytes localized in the pancreas under diabetic condition. Inasmuch as CD4^+^ T-cells appear to mediate T1D via macrophages in the NOD.*scid* adoptive transfer model [25], failure of splenocytes from TSA treated mice to transfer diabetes in NOD.*scid* mice is likely to be due to altered transcription of these genes in CD4^+^ T-cells as well as in macrophages. Although exposure of diabetogenic CD4^+^ T-cells derived from BDC2.5 transgenic mice to TNF-α resulted in the suppression of T cell responses *in vitro* and altered expression of several genes involved in signaling pathways coupled to the T cell receptor, it is not known whether these alterations can impact the manifestation of diabetes [37]. Our observation that TSA treatment resulted in the amelioration of diabetes and repression of a novel set of inflammatory genes is consistent with the possible involvement of these genes in T1D manifestation. Further work is necessary to determine the mechanisms by which the products of these unconventional inflammatory genes can directly or indirectly exert damage to β-cells.

Circumstantial evidence suggests that exaggerated expression of inflammatory cytokines such as IL-6 and TNF-α may contribute to the pathogenesis of several diseases, including T1D [38]. However, the microarray data presented in this report failed to reveal modulation of genes encoding IL-2, IL-4, IL-17, IL-18, TNF-α and iNOS in splenocytes of TSA-treated and cured mice. This may be due to the fact that except *Inos*, all other genes are expressed only after T-cell receptor-mediated activation and therefore will not be discerned by the microarray analysis of un-induced splenocytes. Our previous study indicated that TSA treatment failed to change the expression levels of genes coding for IL-2, IL-4, IL-17, IL-18, and TNF-α in activated T-cells [8]. Both IL-2 and IFN-γ- producing Th1 cells and Th2 cells that produce IL-4 had been implicated in the mediation of T1D in NOD mice [39]–[40]. Whereas adoptive transfer of Th17 cells induced T1D in NOD.*scid* mice, a majority of these cells converted into Th1-like cells in the recipients, suggesting that Th17 cells *per se* may not be the mediators of autoimmune diabetes [41]–[42]. It was shown that Th1 and Th2 cytokine shifts among autoreactive T-cells of NOD mice rendered resistant to diabetes reflect the outcome of protection but not the cause of the disease [43]. Thus, the role of these lymphokines in the manifestation of T1D remains obscure. Several lines of evidence implicate the *Ifng* locus [44] and IFN-γ protein [43], [45] in the alleviation of T1D in NOD mice. Consistently, we also observed the up-regulation of IFN-γ at the mRNA and protein levels by epigenetic modulation [8]. It remains to be determined whether this is the cause or effect of epigenetic processes that lead to the amelioration of T1D.

**Figure 7:**
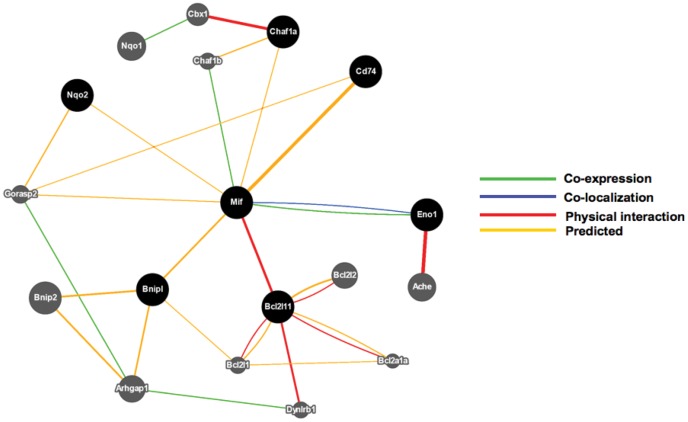
GeneMANIA fast gene function predictions of relationship between *Mif* other interacting genes. *Mif* has been predicted to interact with genes including that encodes its canonical receptor, *Cd74* (indicated by orange lines). *Mif* has been shown to physically interact with pro-apoptotic genes such as *Bnipl* and *Bcl2l11*, which in turn interact with *Bnip2*, *Bcl2l1*, *Bcl2a1a and Bcl2l2*.

Equally impressive is the up-regulation of many genes in mice protected from diabetes by resetting the transcriptional program of splenocytes. The basal level expression of the gene encoding MIF (*Mif*) was similar in the target organ, pancreas of diabetic and cured mice. In contrast, the constitutive level of *Mif* was higher in splenocytes of TSA treated mice and was further enhanced following activation of T lymphocytes through the T-cell receptor. In addition, the gene encoding the MIF receptor, CD74 [33] was also highly expressed in splenocytes, but not in pancreata of protected mice. This is similar to the lower microarray hybridization signal obtained with probe sets designed to interrogate *Cd74* in spleens of prediabetic NOD mice producing anti-insulin antibody [19]. MIF, the first lymphokine reported to be released by activated T lymphocytes [46] has been highly conserved through phylogeny [47]–[48]. In addition to T-cells, MIF is also expressed by other cell types and involved in insulin secretion in beta cell lines, and imparts insulin sensitivity in adipocytes [49]. Ablation of *Mif* gene actually increased blood glucose levels in streptozotocin treated mice, indicating a protective role of MIF against chemically induced diabetes [50]. *Mif* interacts with genes implicated in lymphocyte apoptosis such as *Bnipl* (Bcl2/adenovirus E1B 19 KD interacting protein like) and *Bcl2l11* (Bcl2 like 11-apoptosis facilitator), which can subsequently interact with other pro-apoptotic genes including *Bnip2* (BCL2/adenovirus E1B interacting protein 2), *Bcl2l11* (BCL2-like 11), *Bcl2l2* (BCL2-like 2), and *Bcl2a1a* (B-cell leukemia/lymphoma 2 related protein A1a) (Figure 7). Therefore, it is likely that increased *Mif* expression by TSA treatment may lead to modulation of T-cell apoptosis. In addition, epigenetic modulation may also result in altered self-peptide presentation by CD74, invariant polypeptide associated with MHC class II complex [33], resulting in increased apoptosis of autoreactive T lymphocytes. These mechanisms may explain the abrogation of diabetogenicity of splenocytes in TSA treated mice. Further work is necessary to determine whether epigenetic modulation can reduce the frequency of diabetogenic T-cells and provide protection against T1D by employing deletional mechanisms.

Our microarray analysis indicated the increase in the transcription of many other genes by epigenetic modulation. Notably, spleens of cured mice expressed higher level of *Ermap,* which codes for erythroid adhesion/receptor transmembrane protein in reticulocytes and circulating erythroblasts [27]. In a previous microarray study, higher expression of erythrocyte specific transcripts including *Ermap* was noted in splenocytes of non-diabetic strains of mice in comparison to diabetes-prone NOD mice [15]. Accumulation of increased *Ermap* transcript indicates enhanced erythropoiesis in the red pulp of spleens in drug treated mice. Increased *Ermap* mRNA was also validated by qRT-PCR in pancreata of cured mice, which could be attributed to increased erythroblasts in the circulating blood. It was reported that >200 d old NOD mice develop Coombs’ positive hemolytic anemia, accompanied by decreased hematocrit and splenomegaly [51]. We also observed that similarly aged diabetic NOD mice displayed splenomegaly and low blood volume whereas mice cured of diabetes by TSA treatment displayed increased RBC content and blood volume (unpublished data). These observations indicate that the beneficial effects of histone hyperacetylation accompanying prevention of autoimmune diabetes include enhanced *Ermap* transcription and erythropoiesis in the spleen. Thus, the level of *Ermap* expression may provide a biomarker for the prognosis of T1D.

Another interesting finding is that TSA treatment increased the expression of *Lycat* both in spleens and pancreata, indicating enhanced generation of hematopoietic and endothelial progenitor cells, which may contribute to lymphoid tissue remodeling and regeneration of islets in these mice. Other genes that were selectively up-regulated by epigenetic modification in spleens included the autophagy related gene *Atg4*, and selected anti-oxidant genes, *Prdx1*, *Prdx6*, and *Gpx1,* implying their possible roles, respectively in clearing damaged cells and protection against oxidant induced β-cell cytotoxicity. In addition, *H2afz,* which replaces the canonical histone H2A in a subset of nucleosomes and therefore can modify gene expression [28], was up-regulated under non-diabetic condition. These changes in gene expression may contribute to tissue reorganization in the spleen as well as islet neogeneration in mice cured of T1D by chromatin remodeling.

Over all, our high-throughput analysis has yielded novel data that are consistent with the involvement of epigenetic mechanisms in diabetes manifestation. Copy number variations of genes and non-protein coding genes including microRNAs, which in turn can influence the expression of protein-coding genes, represent other viable epigenetic mechanisms. Nevertheless, the dramatic disease protection afforded by epigenetic modulation illustrates that the regulatory pathways involved in disease manifestation are amenable to manipulation by chromatin remodeling at a fairly late stage in the life of NOD mice (18–24 wk), when the invasive cellular infiltration culminates in the destruction of insulin producing β-cells. This finding is clinically relevant since by the time T1D is diagnosed in patients, most of the β-cells are thought to be destroyed. Therefore, manipulations that can interdict the on-going processes and afford protection against full-blown diabetes will provide significant benefits to T1D patients. NOD mice are the mainstay of immunological manipulations to find a cure for T1D because they mirror human biology remarkably well [52]. However, divergence of mice and humans ∼65 million years ago has imposed many differences between these species that could account for the failure of many therapies that are successful in NOD mice to provide beneficial effects in patients with T1D. Since epigenetic drugs have shown promise in clinical trials for the treatment of cancer without many serious side effects [53], it is reasonable to propose that manipulation of the epigenome using small molecule inhibitors such as TSA may provide benefits to T1D patients with minimal adverse effects. Epigenetic mechanisms represent a paradigm shift in our view of changes in gene expression under diabetic conditions [9]–[10]. Whereas autoantibodies and ‘susceptible’ HLA alleles serve as important biomarkers predictive of T1D risk, gene signatures identified by our transcriptome analysis represent a valuable tool for selecting surrogate biomarkers for the diagnosis as well as prognosis of T1D. Since current treatment of T1D primarily involves long-term, broad-spectrum immunosuppression, targets identified by epigenetic modulation may lead to the development of selective novel therapeutics with minimal adverse side effects.

## Supporting Information

Figure S1 **Hierarchical representation of highly regulated genes.** Shown are the differential expression levels of 164 genes in splenocytes of control-untreated and non-diabetic NOD mice, TSA-treated and cured NOD mice, and untreated-overtly diabetic NOD mice. RNA was pooled from 3–5 mice per experimental group and analyzed by microarray in duplicate. The key for level of expression is shown below the heat map.
(TIFF)

Table S1 **List of primers used for qRT-PCR.** Forward and reverse primers used to interrogate various genes are given. These primer sets were validated following MIQE guidelines.
(PDF)

Table S2 **Highly regulated genes.** The list of highly (up- and down) regulated genes is given along with Affymetrix ID numbers, fold differences between compared groups, and BH p values.
(PDF)

Table S3 **Functional annotation.** The highly regulated genes shown in Table S2 were further analyzed using DAVID bioinformatics tool. The representation of genes under various functional categories is shown.
(PDF)

Table S4 **Genes over-expressed in diabetic mice.** Genes that were over-expressed in overtly diabetic mice and down-regulated by TSA treatment along with BH p values are shown.
(PDF)

Table S5 **Genes under-expressed in diabetic mice.** Genes that were down-regulated due to TSA treatment are shown along with BH p values.
(PDF)
