# Supplementary figures and images for: Transcriptome Analysis of Epigenetically Modulated Genome Indicates Signature Genes in Manifestation of Type 1 Diabetes and Its Prevention in NOD Mice
Source: PLoS One. 2013 Jan 30;8(1):e55074. doi: 10.1371/journal.pone.0055074 (PMC3559426; doi:10.1371/journal.pone.0055074)

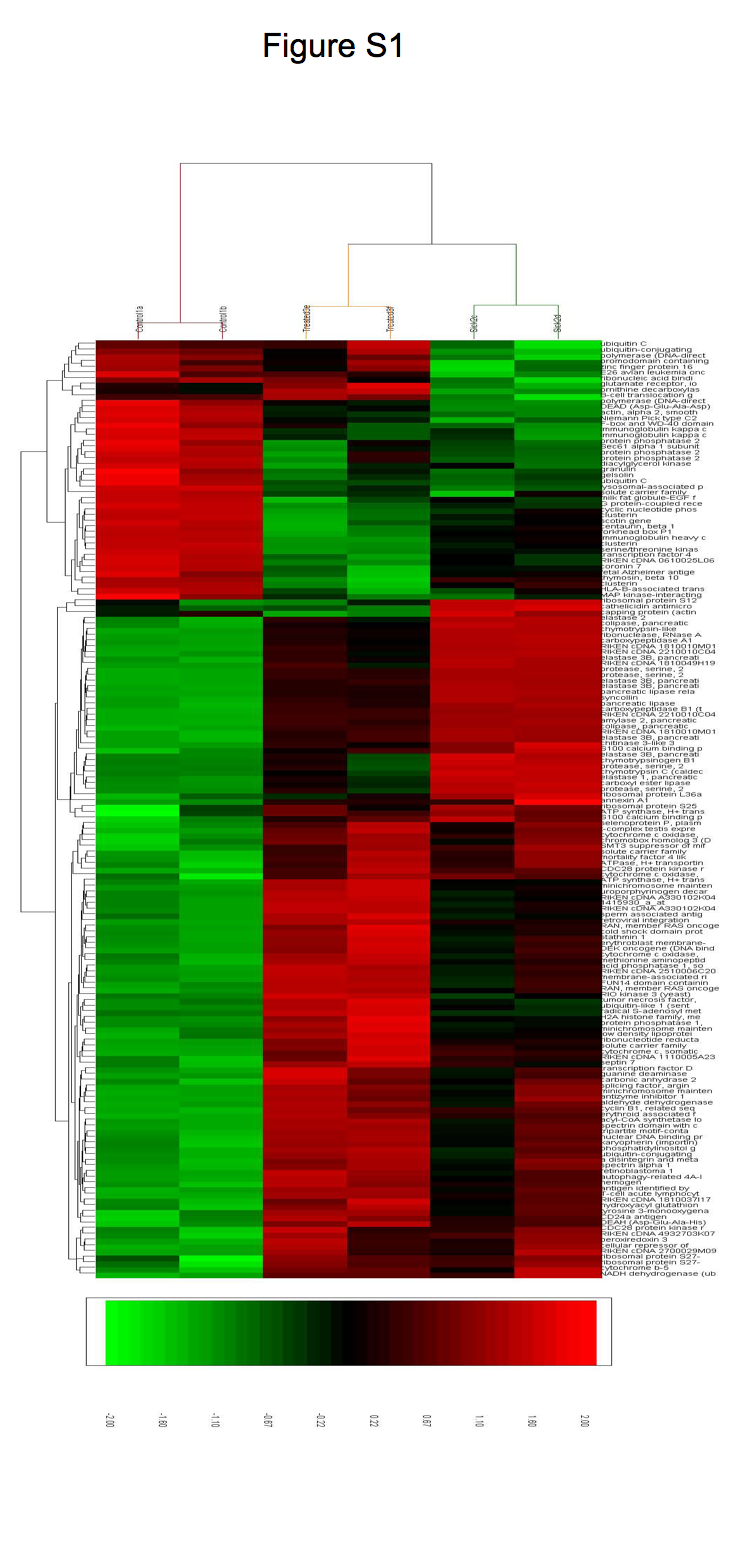

Supplement: Figure S1 — Hierarchical representation of highly regulated genes. Shown are the differential expression levels of 164 genes in splenocytes of control-untreated and non-diabetic NOD mice, TSA-treated and cured NOD mice, and untreated-overtly diabetic NOD mice. RNA was pooled from 3–5 mice per experimental group and analyzed by microarray in duplicate. The key for level of expression is shown below the heat map. (TIFF) [file pone.0055074.s001.tiff]
