# Supplementary material for: Transcriptome Analysis of Epigenetically Modulated Genome Indicates Signature Genes in Manifestation of Type 1 Diabetes and Its Prevention in NOD Mice
Source: PLoS One. 2013 Jan 30;8(1):e55074. doi: 10.1371/journal.pone.0055074 (PMC3559426; doi:10.1371/journal.pone.0055074)
Supplement: Table S1 — List of primers used for qRT-PCR. Forward and reverse primers used to interrogate various genes are given. These primer sets were validated following MIQE guidelines. (PDF) [file pone.0055074.s002.pdf]

Table S1. List of primers used for qRT-PCR

Atg4: Forward-5'-TGCCCTGACTTTCTAAAGGGCTCA-3'

Atg4: Reverse-5'-GCGGTTCAATTCTTGGTGAAGCCA-3'

Ccnb1: Forward-5'-ACCTTGCGGGTTCTAGGGATTGAA-3'

Ccnb1: Reverse-5'-ACAAGGCAGCAAGATCTGGTCTCA-3'

Cd74: Forward-5'-TTGAGTGCAAGCACCTGACTCGTA-3'

Cd74: Reverse-5'-TGGGTCATGTTGCCGTACTTGGTA-3'

Cel: Forward-5'-TGAACATAGCTCTGGGCTTCCCAT-3'

Cel: Reverse-5'-ATCCAGTGTTTGTGTCTCCACCCT-3'

Cela1: Forward-5'-GCCTCCTGAGTGCTGGGATTAAA-3'

Cela1: Reverse-5'-TTCCAACAGGTTGTTGTTGCCCTG-3'

Cela3b: Forward-5'-TCTGACTCAAAGAACTGCCTGCCT-3'

Cela3b: Reverse-5'-AGGAAGGAAGGGTACAGTTTGGCA-3'

Cpa1: Forward-5'-TGGGCAGTGCCTCTTCCTGATATT-3'

Cpa1: Reverse-5'-TCATTGGTGCAGGAGTCCAGATGT-3'

Cpb1: Forward- 5'- TGCGTGCTAGAAATCAGTCCCAGT-3'

Cpb1:Reverse- 5'- TAAATGAGAAAGGCGAGGGCCAGA-3'

Ctrc: Forward-5'-AATTACAGTCCTCGCTGCCATCCT-3'

Ctrc: Reverse-5'-TCTGGAAGAGGCTCAACCAACACA-3'

Ctrl: Forward-5'-ACGCCAACACCATGAACAATGACC-3'

Ctrl: Reverse: 5'- AGACTGGTGAGACTTGTGCTGTGT-3'

Ermap: Forward- 5'-AGGTCCTGAGTTCAATTCCCAGCA-3'

Ermap: Reverse-5'-ATGTTTGCCTGCATGCCAGAAGAG-3'

Gapdh: Forward-5'-TGG TGA AGG TCG GTG TGA AC-3'

Gapdh: Reverse-5'-CCA TGT AGT TGA GGT CAA TGA AGG-3'

Gpx1: Forward-5'-TGCAATCAGTTCGGACACCAGGTA-3'

Gpx1: Reverse-5'-ACCAGGAAGTTGCCCTCCTTGTAT-3'

H2afz: Forward-5'-TCGCTGATCGGGAAGAAAGGACAA-3'

H2afz: Reverse-5'-TCGCTGATCGGGAAGAAAGGACAA-3'

Lycat: Forward-5'-GTGGCATTGCTGGAGACCATGTTT-3'

Lycat: Reverse-5'-TTCATGATGATGACGCTCCGCTCT-3'

March5: Forward-5'-ATGTTATGGAGCGAGCTGACCCTT-3'

March5: Reverse-5'-AGAGACTTCCTCTTGGTTGGCGTT-3'

Mki67: Forward-5'-CCTCATTGCTGGCATGGCGTTTAT-3'

Mki67: Reverse-5'-GAGACTTCCTCTTGGTTGGCGT-3'

Pnlip: Forward-5'-AAGCAATCTAGCCTGACTGGAGCA-3'

Pnlip: Reverse-5'-AAACCCAGGAAGGACACATGAGGT-3'

Pnliprp-1: Forward-5'-AACACAATGCCTTTGCCTACTCGC-3'

Pnliprp-1: Reverse- 5'-TACCATGCTGCCCTTGAACTCAGA-3'

Pnliprp-2: Forward-5'-TGACTTCCTGAAGCCTGTGTGTGA-3'

Pnliprp-2: Reverse-5'-AACATGAAGCCTGGTCTCAGGGAA-3'

Prdx1: Forward-5'-AAGACCTCTGCTTGCTCTGGTCAA-3'

Prdx1: Reverse-5'-AAGTCTTTGGGCTGGATGGACTGA-3'

Prdx6: Forward-5'-AGCAATGCTTTGAGGGAAAGGCTG-3'

Prdx6: Reverse-5'-AATCGGTGCATACCTAGCCACTGT-3'

Stmn1: Forward-5'-AAATCGTTCCAGGGCTTTCCTTGC-3'

Stmn1: Reverse-5'-AAGGCCTGCACCACTTCCTATTCT-3'

Txn1: Forward-5'-TGTGAAGTCAAATGCATGCCGACC-3'

Txn1: Reverse-5'-ATCAAAGGTGTGCATCACCATGCC-3'
