# Supplementary material for: Transcriptome Analysis of Epigenetically Modulated Genome Indicates Signature Genes in Manifestation of Type 1 Diabetes and Its Prevention in NOD Mice
Source: PLoS One. 2013 Jan 30;8(1):e55074. doi: 10.1371/journal.pone.0055074 (PMC3559426; doi:10.1371/journal.pone.0055074)
Supplement: Table S2 — Highly regulated genes. The list of highly (up- and down) regulated genes is given along with Affymetrix ID numbers, fold differences between compared groups, and BH p values. (PDF) [file pone.0055074.s003.pdf]

Table S2. Highly regulated genes

ProbeSet	Acccession	Gene Symbol	Description	Diabetic/Contr	BH.pVal	TSA/Control	BH.pval	Diabetic/TSA	BH.pVal
1457307_at		A330102K04Rik	RIKEN cDNA A	-1.17787586	0.004768369	-3.07264698	1.297E-13	-1.86899272	0.018005029
1420911_a_at		Mfge8	milk fat globul	9.541615505	0.002320811	9.13400082	0.329356348	-1.73262942	0.070482424
1419074_at		Chac2	ChaC, cation t	-1.04430711	0.009630952	-2.11033252	1.15102E-06	-1.12607408	0.662667512
1438092_x_at		H2afz	H2A histone fa	-0.54582275	0.304662095	-1.25050928	0.013839031	-0.84364081	1
1436708_x_at		Mcm4	minichromoso	-0.74340514	0.127218245	-1.56806669	0.000831148	-0.83508516	1
1443849_x_at		Urod	uroporphyrino	-0.52739713	0.209493143	-1.34758078	0.001153317	-0.82253557	0.918108402
1415747_s_at		Riok3	RIO kinase 3 (	-0.24107942	0.949437827	-1.0508642	0.061126764	-0.79026778	1
1438855_x_at		Tnfaip2	tumor necrosis	-0.27959925	0.916724815	-1.0469067	0.063643224	-0.78094567	1
1433540_x_at		LOC100044953 //	protein phospl	-0.40804453	0.654323512	-1.22541072	0.017439888	-0.76753839	1
1436297_a_at		Grina	glutamate rece	0.299621018	0.902644384	-0.43361757	0.689496771	-0.71381492	1
1418909_at		Ermap	erythroblast m	-0.66817687	0.084194043	-1.32901495	0.005944968	-0.70667099	1
1428843_at		March5/	March5/	-0.59393404	0.239672012	-1.18585252	0.023357109	-0.63884228	1
1434120_a_at		Metap2	methionine am	-0.59103176	0.033486643	-1.20499556	0.00066072	-0.62540751	1
1436058_at		Rsad2	radical S-aden	-0.16194716	0.899526928	-0.68250767	0.068394872	-0.6160453	1
1435800_a_at		Csda	cold shock dor	-0.47831312	0.318836077	-1.10095586	0.026158211	-0.60511098	1
1434662_at		Atg4a // LOC100(	autophagy-rela	-0.91748054	0.018896369	-1.53511607	0.001180089	-0.59617446	1
1434437_x_at		Rrm2	ribonucleotide	-0.71740187	0.018345203	-1.34832545	0.001636372	-0.58657956	1
1423883_at		Acsl1	acyl-CoA synth	-0.8293264	0.030014601	-1.3995629	0.003954547	-0.58653069	1
1448670_at		LOC100047012 //	ubiquitin-conju	-0.80763352	0.031767394	-1.27760435	0.011435736	-0.56390655	1
1428316_a_at		Fundc2	FUN14 domair	-0.48854478	0.526147607	-0.9752044	0.101197448	-0.55614779	1
1415849_s_at		Stmn1	stathmin 1	-0.51058306	0.191759702	-0.9656596	0.059999554	-0.53562391	1
1452659_at		Dek	DEK oncogene	-0.65433851	0.001966524	-1.15338661	0.000351379	-0.5269343	1
1435748_at		Gda	guanine deam	-0.60424916	0.184166209	-1.03104175	0.06755909	-0.52277664	1
1437174_at		1110029I05Rik //	transcription fa	-0.74240395	0.100650933	-1.39600148	0.004288396	-0.51134358	1
1458440_at		Specc1	sperm antigen	-0.97057743	0.007211336	-1.44489334	0.002732133	-0.50116017	1
1450721_at		Acp1	acid phosphata	-0.50584119	0.392122579	-0.95906717	0.107276882	-0.49484262	1
1415860_at		Kpna2 // LOC100(	karyopherin (i	-0.98545974	0.001416226	-1.5000914	0.001301694	-0.48852636	1
1416118_at		Trim59	tripartite motif	-0.78710816	0.075489157	-1.24404266	0.016534459	-0.48413713	1
1415930_a_at		Map1lc3b	microtubule-as	-0.32183509	0.765281497	-0.88130397	0.150496175	-0.47531201	1
1435416_x_at		Pigq	phosphatidylin	-0.53110116	0.213293292	-0.99519914	0.067587294	-0.46646027	1
1417850_at		Rb1	retinoblastoma	-0.57189971	0.274684795	-0.91226476	0.139769333	-0.4628964	1
1424143_a_at		Cdt1	chromatin licer	-0.50926385	0.363705697	-1.03453761	0.066706451	-0.46232833	1
1421278_s_at		LOC630963	similar to spec	-0.91771554	0.005901	-1.29432741	0.009777987	-0.4575107	1
1449389_at		Tal1	T-cell acute lyı	-0.92979902	0.005885393	-1.35707027	0.005746499	-0.45614725	1
1436808_x_at		Mcm5	minichromoso	-0.31402451	0.827713051	-0.78864629	0.248977357	-0.44869839	1
1436292_a_at		Oaz1	ornithine deca	0.191370617	0.829490233	-0.21402028	0.818007227	-0.43782974	1
1434578_x_at		LOC100045999 //	RAN, member	-0.34344136	0.766931043	-0.80494886	0.232390366	-0.41761292	1
1450711_at		Brd4	bromodomain	0.457727032	0.587220641	0.124975036	0.960959933	-0.41313446	1
1437278_a_at		Sae2	SUMO1 activat	-0.21764007	0.985383103	-0.63624203	0.432433173	-0.39461324	1
1423090_x_at		LOC100042561 //	SEC61, gamm	-0.50634086	0.357857721	-0.89711533	0.14628763	-0.3699312	1
1424171_a_at		Hagh	hydroxyacyl gl	-0.69371953	0.096646712	-1.07647073	0.052258596	-0.36697434	1
1416468_at		Aldh1a1	aldehyde dehy	-0.6767231	0.127394654	-1.01848198	0.075715015	-0.35794068	1
1426817_at		Mki67	antigen identif	-0.63424064	0.017915583	-1.09398179	0.007334764	-0.35635705	1
1437455_a_at		Btg1 // LOC1000	B-cell transloc	0.298511169	0.744702001	-0.07619699	0.978577855	-0.3546447	1
1448205_at		Ccnb1 // Ccnb1-rs	cyclin B1, rela	-1.16766298	0.000154823	-1.38196552	0.004631008	-0.35114828	1
1416454_s_at		Acta2	actin, alpha 2,	1.349140006	8.2809E-05	0.923310869	0.141264108	-0.34737553	1
1450714_at		Azin1	antizyme inhib	-0.99702193	0.002043402	-1.36804097	0.005266301	-0.34558644	1
1437995_x_at		Septin7/	septin 7	-0.12084281	1	-0.44421145	0.67714411	-0.33520019	1
1448505_at		C1d	nuclear DNA b	-0.42682576	0.637685124	-0.69869569	0.356935696	-0.30075233	1
1449077_at		Eraf	erythroid asso	-0.62531807	4.83866E-08	-0.82539043	1.0244E-05	-0.29883131	1
1428103_at		Adam10	a disintegrin a	-0.7720805	0.046823111	-0.99623687	0.086336051	-0.29214871	1
1439438_a_at		1110005A23Rik //	RIKEN cDNA 1	-0.34903523	0.791715264	-0.62665043	0.443799921	-0.28481336	1
1416476_a_at		Ube2d2	ubiquitin-conju	0.358822596	0.76350747	0.034469122	0.995345252	-0.26223303	1
1448752_at		Car2	carbonic anhyc	-0.78173745	1.48297E-07	-1.06705288	4.56268E-05	-0.26157791	1
1416884_at		Cbx3	chromobox ho	-0.57807493	0.10311295	-0.73489915	0.283679187	-0.25825153	1
1437837_x_at		Poldip3	polymerase (D	0.375364857	0.693480599	0.106598139	0.968957459	-0.25810502	1
1456071_a_at		Cycs // LOC1000	cytochrome c,	-0.54003197	0.210368628	-0.75805691	0.271687941	-0.25359448	1
1416150_a_at		Sfrs3	splicing factor,	-0.60573022	0.19794131	-0.82445975	0.213565751	-0.21790633	1
1448344_at		EG432865 // EG6	ribosomal prot	-0.1465947	1	-0.45197244	0.675754361	-0.21524037	1
1448182_a_at		Cd24a	CD24a antiger	-0.4444565	4.52E-05	-0.64955486	4.58543E-06	-0.20299962	1
1459765_s_at		Sf1	Splicing factor	0.247209054	0.781444148	0.078974709	0.781444148	-0.20104482	1
1417458_s_at	Cks2 // LOC10	CDC28 protein kinase regulatory		-1.09934561	0.001117165	-1.23838762	0.017129509	-0.17481415	1
1437468_x_at		Fbxw11	F-box and WD	0.461667833	0.523207553	0.314715413	0.816562691	-0.16287396	1
1437027_x_at		LOC100040661 //	ribonucleic aci	0.23617944	0.705379463	0.09635649	0.965607371	-0.16238952	0.972832475
1448513_a_at		Npc2	Niemann Pick	0.355160741	0.713372952	0.251679398	0.873364187	-0.14412698	1
1437773_x_at		Ddx17	DEAD (Asp-Glu	0.349995032	0.7101338	0.236358826	0.883558634	-0.1402045	1
1424365_at		1810037I17Rik	RIKEN cDNA 1	-0.28218061	0.900391134	-0.51206222	0.59224277	-0.13557338	1
1428116_a_at		Dynlt1 // LOC100	dynein light ch	-0.36575674	0.732623745	-0.50218588	0.604922287	-0.13297862	1
1415948_at		Creg1	cellular repres	-0.88280432	0.020586857	-0.90053512	0.156837884	-0.1299945	1
1436905_x_at		Laptm5	lysosomal-asso	0.78596615	0.021975329	0.620183817	0.439914647	-0.12835983	1
1456743_x_at	LOC433261 //	mortality factor 4	like 2 // simila	-0.40981748	0.566297474	-0.4684626	0.644268908	-0.11283086	1
1438383_x_at		Ppp2r1a	protein phospl	0.567826477	0.190653878	0.461697913	0.644672191	-0.11071787	1
1416727_a_at		Cyb5	cytochrome b-	-0.57482083	0.262257784	-0.67548986	0.380626965	-0.11017856	1
1452417_x_at		2010205A11Rik //	immunoglobul	0.74504298	9.0958E-06	0.675381047	0.16160058	-0.10742574	1
1416144_a_at		Dhx15	DEAH (Asp-Glu	-0.40291184	0.623634174	-0.54687173	0.545964938	-0.10269826	1
1460590_s_at		LOC100039786 //	tyrosine 3-mor	-0.28160536	0.878977496	-0.40405482	0.721317783	-0.10100329	1
1452141_a_at		Sepp1	selenoprotein	-0.39174632	0.410085592	-0.47842818	0.596747251	-0.1005756	1
1435429_x_at		Rps27l	ribosomal prot	-0.64053669	0.192358944	-0.70355615	0.350624336	-0.09163985	1
1428212_x_at		EG665562 // EG6	ribosomal prot	-0.03697755	1	-0.1314281	0.794829288	-0.08932161	1
1438559_x_at		Slc44a2	solute carrier f	0.626934398	0.135241497	0.546999068	0.538226719	-0.08423014	1
1438991_x_at		Ppp2r1a	protein phospl	0.550820952	0.216550183	0.473533948	0.631642687	-0.06675312	1
1456567_x_at		Grn	granulin	0.333245546	0.800834029	0.193451087	0.917202564	-0.06143206	1
1416292_at		Prdx3	peroxiredoxin	-0.73149228	0.079875732	-0.7892772	0.252732198	-0.0510598	1

Table S2. Highly regulated genes

1455929_x_at	Ppp2r1a	protein phosph	0.462381706	0.475932084	0.490476579	0.616524346	-0.043082	1
1416189_a_at	Sec61a1	Sec61 alpha 1	0.41451427	0.666158205	0.494913952	0.619163635	-0.03865711	1
1423254_x_at	Rps27l	ribosomal prot	-0.68719359	0.13606052	-0.68173114	0.378425324	-0.02546945	1
1437171_x_at	Gsn	gelsolin	0.619827008	0.223719883	0.628908833	0.442332624	-0.01882754	1
1438902_a_at	Hsp90aa1	heat shock pro	-0.63036317	0.044414395	-0.65930076	0.373102166	-0.0117951	1
1417061_at	Slc40a1	solute carrier f	-0.38354031	0.565533277	-0.48562781	0.616840237	-0.00790216	1
1448112_at	Cox7c // LOC100(	cytochrome c	-0.33697947	0.79817123	-0.37762702	0.75373698	-0.00496104	1
1437666_x_at	Ubc	Ubiquitin C	0.247686482	0.850901303	0.245015894	0.869251074	-0.00249781	1
1437666_x_at	Ubc	ubiquitin C	0.247686482	0.247686482	0.245015894	0.869251074	-0.00249781	1
1418199_at	Hemgn	hemogen	-1.28148714	0.000405314	-0.55339438	0.004699982	4.06781E-06	1
1418300_a_at	Mknk2	MAP kinase-int	0.3609485	0.716621638	0.419664205	0.703458954	0.015797264	1
1456349_x_at	Sumo1	SMT3 suppres:	-0.16546629	1	-0.24414615	0.879816138	0.031041914	1
1427660_x_at	Cr1 // ENSMUSG(	immunoglobul	0.710536032	3.37845E-11	0.570636855	0.074630425	0.03181802	1
1424085_at	Ndufa4	NADH dehydro	-0.52777314	0.347789832	-0.40297904	0.722429311	0.067156232	1
1416269_at	Atp5j2	ATP synthase,	-0.65861884	0.149266471	-0.57317173	0.511072375	0.078232094	1
1452877_at	2700029M09Rik	RIKEN cDNA 2	-0.61486411	0.211916179	-0.68843829	0.36648596	0.093132462	1
1437436_s_at	Grk6	G protein-coup	0.353526079	0.76819685	0.486332812	0.626267539	0.104288925	1
1451068_s_at	Rps25	ribosomal prot	-0.41061088	0.096210216	-0.3097307	0.730225318	0.104388436	1
1452169_a_at	Dgkz	diacylglycerol	0.897704873	0.015205092	1.081007188	0.054349139	0.12187533	1
1426725_s_at	Ets1	E26 avian leuk	0.332233691	0.742648373	0.1	0.742648373	0.13	1
1419394_s_at	S100a8	S100 calcium	-0.60477098	4.60136E-07	-0.42006909	0.310041444	0.132475488	1
1437984_x_at	Bat1a	HLA-B-associa	0.412465942	0.578800115	0.627029038	0.437932038	0.165768983	1
1437503_a_at	Scotin	scotin gene	0.37525461	0.157243787	0.599773893	0.332995043	0.204048755	1
1437185_s_at	LOC100042319 //	thymosin, beta	0.140150181	0.015888871	0.417024221	0.041100808	0.262454447	1
1437689_x_at	Clu // LOC100046	clusterin // sin	0.528436762	0.231019531	0.880943043	0.154039622	0.287788658	1
1456377_x_at	Limd2 // LOC6323	LIM domain co	0.610565565	0.057525571	0.886374391	0.14276588	0.296502594	1
1437341_x_at	Cnp	2',3'-cyclic nuc	0.503234384	0.371992297	0.787843197	0.244913606	0.30403254	1
1434148_at	Tcf4	transcription fa	0.473569023	0.566297474	0.889611001	0.176785928	0.331340667	1
1448344_at	EG432865 // EG6	ribosomal prot	-0.33219297	0.784399989	0.005382891	1	0.341005434	1
1456615_a_at	Bptf	bromodomain	0.405344654	0.661805569	0.846831822	0.204229691	0.344368169	1
1437524_x_at	Coro7	coronin 7	0.53534689	0.440336623	0.908240314	0.169204331	0.350563026	1
1435222_at	Foxp1	forkhead box I	0.542158451	0.205850604	0.910108331	0.13129192	0.35641191	1
1448213_at	Anxa1	annexin A1	-0.92517369	0.511468942	-0.57543477	1	0.386000351	0.081258638
1423057_at	Capza2	capping protei	-0.15942894	1	0.190479564	0.918730009	0.390646156	1
1419764_at	Chi3l3	chitinase 3-like	-0.9512685	0.003058062	-0.51804856	0.583656165	0.441027372	1
1434873_a_at	Centb1 // LOC100	centaurin, beta	0.642549927	0.190220069	1.263685601	0.016140357	0.604071204	1
1419691_at	Camp	cathelicidin an	-0.57704003	0.158702625	0.101802881	0.97057958	0.631288369	1
1447806_s_at	Srpk3	Srpk3	0.991181078	0.010564533	1.721562803	0.000545504	0.721175941	1
1437837_x_at	Poldip3	polymerase (D	0.375364857	0.693480599	-0.25810502	0.968957459	0.772024226	1
1427503_at	AI324046	expressed seq	2.303055867	4.21343E-11	4.333416398	0	2.01696177	0.036863547
1434137_x_at	1810010M01Rik	RIKEN cDNA 1	-6.09704312	0	-4.19389938	0	2.187171419	2.19662E-05
1428102_at	Cpb1	carboxypeptida	-7.36203501	0	-4.66146221	0	2.465626266	2.20215E-06
1437326_x_at	Ela3 // LOC63841	elastase 3, pa	-6.68560308	0	-4.38319174	0	2.520184407	1.24747E-07
1438612_a_at	Clps	colipase, panc	-7.70327033	0	-5.33614277	0	2.533103933	6.74585E-08
1416523_at	Rnase1	ribonuclease, I	-5.67747187	0	-2.90894099	3.55361E-09	2.624736503	1.23055E-05
1416055_at	1810008N23Rik /,	amylase 2, pa	-8.18943599	0	-5.83872907	0	2.735658965	1.72004E-10
1431763_a_at	Ctrl	chymotrypsin-	-6.03601304	0	-3.2322357	4.4111E-13	2.749515022	1.1962E-07
1433431_at	Pnlip	pancreatic lipa	-7.59196474	0	-4.99546492	0	2.809383328	4.5101E-10
1423693_at	Ela1	elastase 1, pa	-5.20208503	0	-1.99072268	1.6548E-05	3.165983777	8.88909E-12
1415883_a_at	Ela3	elastase 3, pa	-6.94467037	0	-3.97840451	0	3.226241947	2.89381E-10
1434747_at	Ctrc	chymotrypsin	-4.78681604	0	-1.45287328	0.022010333	3.23324166	6.92396E-10
1428062_at	Cpa1	carboxypeptida	-7.13520514	0	-3.60002173	5.9E-15	3.311683848	5.33345E-11
1422435_at	2210010C04Rik	RIKEN cDNA 2	-7.25488739	0	-3.90847038	0	3.5191692	0
1417257_at	Cel	carboxyl ester	-6.22958237	0	10/29/201110	0.000201226	3.555426229	1.72004E-10
1448281_a_at	RP23-395H4.4	elastase 2A	-6.82339485	0	-3.03084049	8.93959E-11	3.594261138	5.71441E-12
1417682_a_at	Prss2	protease, seri	-5.4916247	0	-1.75078596	0.000589276	3.838999227	0
1448220_at	Ctrb1	chymotrypsinc	-6.06435384	0	-2.1773934	2.4155E-05	4.211742679	0
