# Supplementary material for: Transcriptome Analysis of Epigenetically Modulated Genome Indicates Signature Genes in Manifestation of Type 1 Diabetes and Its Prevention in NOD Mice
Source: PLoS One. 2013 Jan 30;8(1):e55074. doi: 10.1371/journal.pone.0055074 (PMC3559426; doi:10.1371/journal.pone.0055074)
Supplement: Table S3 — Functional annotation. The highly regulated genes shown in Table S2 were further analyzed using DAVID bioinformatics tool. The representation of genes under various functional categories is shown. (PDF) [file pone.0055074.s004.pdf]

Table S3. Functional annotation

Term	Count	%	P-value
Phosphoprotein	60	44.7	6.4E-2
Acetylation	50	37.3	2.5E-11
Cytoplasm	32	22.8	7.2E-2
Non-membrane-bounded organelle	24	17.9	1.2E-2
Hydrolase	23	17.2	2.4E-3
Proteolysis	20	14.9	7.5E-4
Extracellular region	20	14.9	2.3E-2
Secreted	19	14.1	1.7E-2
ATP binding	16	11.9	8.2E-2
Peptidase activity	13	9.7	5.8E-3
Protease	12	8.9	1.3E-3
Vesicle	11	8.2	6.5E-3
Cell cycle	11	8.2	4.5E-2
Macromolecule catabolic process	11	8.2	5.5E-2
Cytoplasmic vesicle	11	8.2	5.7E-3
Organelle envelope	10	7.5	2.3
Envelope	10	7.5	2.3E-2
Endopeptidase activity	10	7.5	7.8E-3
Ribonucleoprotein complex	10	7.5	7.9E-3

Analyzed using DAVID Bioinformatics Tool. Note that some of the genes are represented in multiple categories.
