# Supplementary material for: Transcriptome Analysis of Epigenetically Modulated Genome Indicates Signature Genes in Manifestation of Type 1 Diabetes and Its Prevention in NOD Mice
Source: PLoS One. 2013 Jan 30;8(1):e55074. doi: 10.1371/journal.pone.0055074 (PMC3559426; doi:10.1371/journal.pone.0055074)
Supplement: Table S4 — Genes over-expressed in diabetic mice. Genes that were over-expressed in overtly diabetic mice and down-regulated by TSA treatment along with BH p values are shown. (PDF) [file pone.0055074.s005.pdf]

Table S4. Genes over-expressed in diabetic mice

ProbeSet	Gene Syml	Description	Diabetic/Control	BH.pVal	TSA/Control	BH.pval	Diabetic/TSA	BH.pVal
1415777_at	Pnliprp1	pancreatic lipa	30.64150134	0	10.19641159	1.83E-12	5.004263188	0.000676
1448220_at	Ctrb1	chymotrypsino	-6.064353836	0	-2.177393405	2.42E-05	4.211742679	0
1417682_a_	Prss2	protease, serin	-5.491624704	0	-1.750785961	0.000589	3.838999227	0
1448281_a_	RP23-395H4	elastase 2A	-6.823394855	0	-3.030840489	8.94E-11	3.594261138	5.71E-12
1417257_at	Cel	carboxyl ester	-6.229582374	0	-2.041850333	0.000201	3.555426229	1.72E-10
1428062_at	Cpa1	carboxypeptida	-7.135205141	0	-3.600021729	5.9E-15	3.311683848	5.33E-11
1434747_at	Ctrc	chymotrypsin C	-4.786816043	0	-1.452873277	0.02201	3.23324166	6.92E-10
1423693_at	Ela1	elastase 1, par	-5.202085026	0	-1.990722681	1.65E-05	3.165983777	8.89E-12
1433431_at	Pnlip	pancreatic lipa	-7.59196474	0	-4.995464917	0	2.809383328	4.51E-10
1431763_a_	Ctrl	chymotrypsin-l	-6.036013044	0	-3.232235697	4.41E-13	2.749515022	1.2E-07
1416055_at	Amy2	amylase 2, par	-8.189435989	0	-5.838729072	0	2.735658965	1.72E-10
1416523_at	Rnase1	ribonuclease, R	-5.677471872	0	-2.908940994	3.55E-09	2.624736503	1.23E-05
1438612_a_	Clps	colipase, pancr	-7.703270328	0	-5.336142771	0	2.533103933	6.75E-08
1437326_x_	Ela3	elastase 3, par	-6.685603085	0	-4.383191735	0	2.520184407	1.25E-07
1428102_at	Cpb1	carboxypeptida	-7.362035012	0	-4.661462214	0	2.465626266	2.2E-06
1434137_x_	1810010M0	RIKEN cDNA 1	-6.097043119	0	-4.19389938	0	2.187171419	2.2E-05
1427503_at	AI324046	expressed seq	2.303055867	4.21E-11	4.333416398	0	2.01696177	0.036864
