# Supplementary material for: Transcriptome Analysis of Epigenetically Modulated Genome Indicates Signature Genes in Manifestation of Type 1 Diabetes and Its Prevention in NOD Mice
Source: PLoS One. 2013 Jan 30;8(1):e55074. doi: 10.1371/journal.pone.0055074 (PMC3559426; doi:10.1371/journal.pone.0055074)
Supplement: Table S5 — Genes under-expressed in diabetic mice. Genes that were down-regulated due to TSA treatment are shown along with BH p values. (PDF) [file pone.0055074.s006.pdf]

Table S5. Genes under-expressed in diabetic mice and upregulated by TSA treatment

ProbeSet	Gene Symbol	Description	Diabetic/Contr	BH.pVal	TSA/Control	BH.pval	Diabetic/TSA	BH.pVal
1418909_at	Ermap	erythroblast mer	-0.668176868	0.084194043	-1.329014953	0.005944968	-0.706670988	1
1438092_x_at	H2afz	H2A histone fam	-0.545822746	0.304662095	-1.25050928	0.013839031	-0.843640812	1
1428843_at	March 5//	March5//	-0.593934037	0.239672012	-1.185852519	0.023357109	-0.638842284	1
1448205_at	Ccnb1	cyclin B1, relate	-1.16766298	0.000154823	-1.381965521	0.004631008	-0.351148278	1
1426817_at	Mki67	antigen identifie	-0.634240642	0.017915583	-1.093981791	0.007334764	-0.356357046	1
1434662_at	Atg4a	similar to autopl	-0.917480539	0.018896369	-1.535116075	0.001180089	-0.596174456	1
1415849_s_at	Stmn1	stathmin 1	-0.510583057	0.191759702	-0.965659595	0.059999554	-0.535623908	1
1434120_a_at	Metap2	methionine amir	-0.591031755	0.033486643	-1.204995558	0.00066072	-0.625407514	1
1436058_at	Rsad2	radical S-adenos	-0.161947163	0.899526928	-0.682507671	0.068394872	-0.616045299	1
1435800_a_at	Csda	cold shock doma	-0.478313118	0.318836077	-1.100955858	0.026158211	-0.605110983	1
1436708_x_at	Mcm4	minichromosome	-0.743405136	0.127218245	-1.568066691	0.000831148	-0.835085161	1
1434437_x_at	Rrm2	ribonucleotide re	-0.717401872	0.018345203	-1.348325447	0.001636372	-0.586579563	1
1423883_at	Acsl1	acyl-CoA synthe	-0.829326395	0.030014601	-1.399562904	0.003954547	-0.586530695	1
1428316_a_at	Fundc2	FUN14 domain c	-0.488544775	0.526147607	-0.975204403	0.101197448	-0.556147786	1
1452659_at	Dek	DEK oncogene (I	-0.654338512	0.001966524	-1.153386606	0.000351379	-0.790267776	1
1435748_at	Gda	guanine deamina	-0.604249161	0.184166209	-1.031041754	0.06755909	-0.101003294	1
1437174_at	Tfdp2	transcription fact	-0.74240395	0.100650933	-1.396001484	0.004288396	0.031041914	1
1458440_at	Specc1	sperm antigen w	-0.970577433	0.007211336	-1.44489334	0.002732133	0.093132462	1
1450721_at	Acp1	acid phosphatas	-0.505841188	0.392122579	-0.959067174	0.107276882	-0.135573384	1
1415860_at	Kpna2	karyopherin (imp	-0.985459736	0.001416226	-1.500091396	0.001301694	-1.868992718	0.018005029
1416118_at	Trim59	tripartite motif-c	-0.787108162	0.075489157	-1.244042658	0.016534459	-1.791593383	0.026765019
1415930_a_at	Map1lc3b	microtubule-ass	-0.321835089	0.765281497	-0.881303968	0.150496175	-0.284813358	1
1435416_x_at	Pigq	phosphatidylinos	-0.531101164	0.213293292	-0.995199141	0.067587294	-0.526934299	1
1417850_at	Rb1	retinoblastoma 1	-0.571899714	0.274684795	-0.912264761	0.139769333	-0.522776642	1
1424143_a_at	Cdt1	chromatin licens	-0.509263845	0.363705697	-1.034537611	0.066706451	-0.51134358	1
1421278_s_at	LOC630963	similar to spectr	-0.917715537	0.005901	-1.294327414	0.009777987	-0.501160173	1
1449389_at	Tal1	T-cell acute lymp	-0.929799017	0.005885393	-1.357070267	0.005746499	-0.494842619	1
1436808_x_at	Mcm5	minichromosome	-0.314024512	0.827713051	-0.788646288	0.248977357	-0.488526361	1
1436292_a_at	Oaz1	ornithine decarb	0.191370617	0.829490233	-0.214020284	0.818007227	-0.484137134	1
1434578_x_at	Ran // Rasl2-9	RAN, member R	-0.343441361	0.766931043	-0.804948856	0.232390366	-0.47531201	1
1450711_at	Brd4	bromodomain cc	0.457727032	0.587220641	0.124975036	0.960959933	-0.466460269	1
1437278_a_at	Sae2	SUMO1 activatin	-0.217640074	0.985383103	-0.636242027	0.432433173	-0.462896403	1
1423090_x_at	Sec61g	SEC61, gamma s	-0.506340863	0.357857721	-0.89711533	0.14628763	-0.462328334	1
1424171_a_at	Hagh	hydroxyacyl glut	-0.693719529	0.096646712	-1.076470725	0.052258596	-0.457510704	1
1416468_at	Aldh1a1	aldehyde dehydr	-0.676723101	0.127394654	-1.01848198	0.075715015	-0.456147249	1
1437455_a_at	Btg1	B-cell translocati	0.298511169	0.744702001	-0.076196985	0.978577855	-0.448698388	1
1416454_s_at	Acta2	actin, alpha 2, s	1.349140006	8.2809E-05	0.923310869	0.141264108	-0.437829736	1
1450714_at	Azin1	antizyme inhibit	-0.99702193	0.002043402	-1.36804097	0.005266301	-0.417612925	1
1437995_x_at	Sept7//	septin 7	-0.120842807	1	-0.444211446	0.67714411	-0.413134465	1
1448505_at	C1d	nuclear DNA bin	-0.426825756	0.637685124	-0.698695691	0.356935696	-0.394613238	1
1449077_at	Eraf	erythroid associ	-0.625318068	4.83866E-08	-0.82539043	1.0244E-05	-0.369931203	1
1428103_at	Adam10	a disintegrin and	-0.772080496	0.046823111	-0.996236869	0.086336051	-0.366974343	1
1416476_a_at	Ube2d2	ubiquitin-conjug	0.358822596	0.76350747	0.034469122	0.995345252	-0.357940675	1
1448752_at	Car2	carbonic anhydr	-0.781737455	1.48297E-07	-1.067052884	4.56268E-05	-0.354644701	1
1416884_at	Cbx3	chromobox hom	-0.578074927	0.10311295	-0.734899149	0.283679187	-0.347375526	1
1437837_x_at	Poldip3	polymerase (DN	0.375364857	0.693480599	0.106598139	0.968957459	-0.34558644	1
1456071_a_at	Cycs	cytochrome c, s	-0.540031973	0.210368628	-0.758056906	0.271687941	-0.33520019	1
1416150_a_at	Sfrs3	splicing factor, a	-0.605730221	0.19794131	-0.824459746	0.213565751	-0.300752333	1
1448344_at	Rps12	ribosomal protei	-0.146594696	1	-0.451972444	0.675754361	-0.298831313	1
1448182_a_at	Cd24a	CD24a antigen	-0.444456504	4.52E-05	-0.649554861	4.58543E-06	-0.292148709	1
1459765_s_at	Sf1	Splicing factor 1	0.247209054	0.781444148	0.078974709	0.781444148	-0.26223303	1
1417458_s_at	CDC28 protein kinase regulatory		-1.099345614	0.001117165	-1.238387616	0.017129509	-0.261577913	1
1417457_at	Cks2	CDC28 protein k	-1.08447513	0.000122035	-1.381268381	0.004380981	-0.258251528	1
1437468_x_at	Fbxw11	F-box and WD-4	0.461667833	0.523207553	0.314715413	0.816562691	-0.25810502	1
1448513_a_at	Npc2	Niemann Pick ty	0.355160741	0.713372952	0.251679398	0.873364187	-0.253594483	1
1437773_x_at	Ddx17	DEAD (Asp-Glu-	0.349995032	0.7101338	0.236358826	0.883558634	-0.217906334	1
1428116_a_at	Dynlt1	dynein light chai	-0.365756742	0.732623745	-0.50218588	0.604922287	-0.21524037	1
1415948_at	Creg1	cellular represso	-0.882804322	0.020586857	-0.900535125	0.156837884	-0.202999621	1
1436905_x_at	Laptm5	lysosomal-assoc	0.78596615	0.021975329	0.620183817	0.439914647	-0.201044823	1
1456743_x_at	mortality factor 4 like 2 // similar		-0.409817482	0.566297474	-0.468462599	0.644268908	-0.174814153	1
1438383_x_at	Ppp2r1a	protein phospha	0.567826477	0.190653878	0.461697913	0.644672191	-0.164293554	1
1416727_a_at	Cyb5	cytochrome b-5	-0.574820825	0.262257784	-0.675489862	0.380626965	-0.162873959	1
1452417_x_at	2010205A11Rik	immunoglobulin	0.74504298	9.0958E-06	0.675381047	0.16160058	-0.144126977	1
1416144_a_at	Dhx15	DEAH (Asp-Glu-	-0.402911836	0.623634174	-0.54687173	0.545964938	-0.140204496	1
1452141_a_at	Sepp1	selenoprotein P,	-0.391746319	0.410085592	-0.47842818	0.596747251	-0.13297862	1
1435429_x_at	Rps27l	ribosomal protei	-0.640536693	0.192358944	-0.703556154	0.350624336	-0.129994501	1
1428212_x_at	EG665562	ribosomal protei	-0.036977545	1	-0.131428098	0.794829288	-0.128359833	1
1438559_x_at	Slc44a2	solute carrier fa	0.626934398	0.135241497	0.546999068	0.538226719	-0.11283086	1
1438991_x_at	Ppp2r1a	protein phospha	0.550820952	0.216550183	0.473533948	0.631642687	-0.110717868	1
1456567_x_at	Grn	granulin	0.333245546	0.800834029	0.193451087	0.917202564	-0.110178561	1
1416292_at	Prdx3	peroxiredoxin 3	-0.731492276	0.079875732	-0.789277199	0.252732198	-0.107425744	1
1455929_x_at	Ppp2r1a	protein phospha	0.462381706	0.475932084	0.490476579	0.616524346	-0.102698264	1
1416189_a_at	Sec61a1	Sec61 alpha 1 s	0.41451427	0.666158205	0.494913952	0.619163635	-0.100575597	1
1423254_x_at	Rps27l	ribosomal protei	-0.687193589	0.13606052	-0.681731138	0.378425324	-0.091639845	1
1437171_x_at	Gsn	gelsolin	0.619827008	0.223719883	0.628908833	0.442332624	-0.089321609	1
1438902_a_at	Hsp90aa1	heat shock prote	-0.630363169	0.044414395	-0.659300758	0.373102166	-0.084230138	1
1417061_at	Slc40a1	solute carrier fa	-0.383540308	0.565533277	-0.485627812	0.616840237	-0.066753117	1
1448112_at	Cox7c	cytochrome c ox	-0.336979465	0.79817123	-0.377627019	0.75373698	-0.061432061	1
1437666_x_at	Ubc	Ubiquitin C	0.247686482	0.850901303	0.245015894	0.869251074	-0.051059798	1
1418199_at	Hemgn	hemogen	-1.281487136	0.000405314	-0.553394381	0.004699982	-0.043081995	1

Table S5. Genes under-expressed in diabetic mice and upregulated by TSA treatment

1418300_a_at	Mknk2	MAP kinase-inter	0.3609485	0.716621638	0.419664205	0.703458954	-0.038657106	1
1427660_x_at	Cr1	immunoglobulin	0.710536032	3.37845E-11	0.570636855	0.074630425	-0.025469447	1
1424085_at	Ndufa4	NADH dehydroge	-0.527773145	0.347789832	-0.402979041	0.722429311	-0.018827541	1
1416269_at	Atp5j2	ATP synthase, H	-0.658618836	0.149266471	-0.573171727	0.511072375	-0.011795101	1
1437436_s_at	Grk6	G protein-couple	0.353526079	0.76819685	0.486332812	0.626267539	-0.007902156	1
1451068_s_at	Rps25	ribosomal protei	-0.410610882	0.096210216	-0.309730701	0.730225318	-0.004961037	1
1452169_a_at	Dgkz	diacylglycerol kir	0.897704873	0.015205092	1.081007188	0.054349139	-0.002497806	1
1426725_s_at	Ets1	E26 avian leuker	0.332233691	0.742648373	0.1	0.742648373	4.06781E-06	1
1419394_s_at	S100a8	S100 calcium bir	-0.604770983	4.60136E-07	-0.420069086	0.310041444	0.015797264	1
1437984_x_at	Bat1a	HLA-B-associate	0.412465942	0.578800115	0.627029038	0.437932038	0.03181802	1
1437503_a_at	Scotin	scotin gene	0.37525461	0.157243787	0.599773893	0.332995043	0.067156232	1
1437341_x_at	Cnp	2',3'-cyclic nucle	0.503234384	0.371992297	0.787843197	0.244913606	0.078232094	1
1434148_at	Tcf4	transcription fact	0.473569023	0.566297474	0.889611001	0.176785928	0.104288925	1
1448344_at	EG432865	ribosomal protei	-0.332192971	0.784399989	0.005382891	1	0.104388436	1
1456615_a_at	Bptf	bromodomain PH	0.405344654	0.661805569	0.846831822	0.204229691	0.12187533	1
1437524_x_at	Coro7	coronin 7	0.53534689	0.440336623	0.908240314	0.169204331	0.13	1
1435222_at	Foxp1	forkhead box P1	0.542158451	0.205850604	0.910108331	0.13129192	0.132475488	1
1437185_s_at	Tmsb10	thymosin, beta 1	0.140150181	0.015888871	0.417024221	0.041100808	0.165768983	1
1423057_at	Capza2	capping protein	-0.159428941	1	0.190479564	0.918730009	0.204048755	1
1419764_at	Chi3l3	chitinase 3-like 3	-0.951268501	0.003058062	-0.518048557	0.583656165	0.30403254	1
1434873_a_at	Centb1	centaurin, beta 1	0.642549927	0.190220069	1.263685601	0.016140357	0.331340667	1
1419691_at	Camp	cathelicidin antir	-0.57704003	0.158702625	0.101802881	0.97057958	0.341005434	1
1447806_s_at	Srpk3	Srpk3	0.991181078	0.010564533	1.721562803	0.000545504	0.344368169	1
1437837_x_at	Poldip3	polymerase (DNA	0.375364857	0.693480599	-0.25810502	0.968957459	0.350563026	1
1437689_x_at	Clu	clusterin // simil	0.528436762	0.231019531	0.880943043	0.154039622	0.35641191	1
1456377_x_at	Limd2	LIM domain cont	0.610565565	0.057525571	0.886374391	0.14276588	0.262454447	1
1448670_at	Ube2e3	ubiquitin-conjug	-0.807633517	0.031767394	-1.277604348	0.011435736	0.390646156	1
1438855_x_at	Tnfaip2	tumor necrosis f	-0.279599252	0.916724815	-1.046906696	0.063643224	0.441027372	1
1433540_x_at	Ppp1cb	protein phosphat	-0.408044529	0.654323512	-1.225410723	0.017439888	0.604071204	1
1436297_a_at	Grina	glutamate recep	0.299621018	0.902644384	-0.433617571	0.689496771	0.631288369	1
1415747_s_at	Riok3	RIO kinase 3 (ye	-0.241079424	0.949437827	-1.050864203	0.061126764	0.721175941	1
1460590_s_at	LOC100039786/	tyrosine 3-mono	-0.28160536	0.878977496	-0.404054819	0.721317783	0.772024226	1
1456349_x_at	Sumo1	SMT3 suppresso	-0.165466287	1	-0.244146148	0.879816138	0.287788658	1
1452877_at	2700029M09Rik	RIKEN cDNA 270	-0.614864112	0.211916179	-0.688438294	0.36648596	0.296502594	1
1424365_at	1810037I17Rik	RIKEN cDNA 181	-0.282180615	0.900391134	-0.512062221	0.59224277	-0.563906545	1
1457307_at	A330102K04Rik	RIKEN cDNA A33	-1.177875862	0.004768369	-3.072646981	1.297E-13	-0.78094567	1
1439438_a_at	1110005A23Rik	RIKEN cDNA 111	-0.349035227	0.791715264	-0.626650426	0.443799921	-0.713814917	1
